# Supplementary material for: Antenatal corticosteroid administration and early school age child development: A regression discontinuity study in British Columbia, Canada
Source: PLoS Med. 2020 Dec 7;17(12):e1003435. doi: 10.1371/journal.pmed.1003435 (PMC7721186; doi:10.1371/journal.pmed.1003435)

**S1 Fig** Schematic of the regression discontinuity design assuming antenatal corticosteroids increases the risk of adverse developmental outcome (hypothetical data). The vertical dashed line indicates the upper gestational age cut-off for routine administration of antenatal corticosteroids (33+6 weeks’ gestation). The terms ‘β1’ and ‘β2’ estimate the underlying trend in risk of adverse neurodevelopmental outcomes associated with gestational age (as risks of most adverse outcomes decrease with advancing gestational age) before and after the cut-off, respectively. The term ‘β3’ is the primary estimate of interest, which estimates if there is a “jump” or level change in risk at the gestational age at which corticosteroids stop being administered routinely (i.e., at the point of discontinuity in corticosteroid treatment practices). If antenatal corticosteroid administration causes adverse neurodevelopmental outcomes, we would expect a *decrease* in risk at 34+0 weeks, reflecting that risks dropped once fetuses were no longer exposed to antenatal corticosteroids.


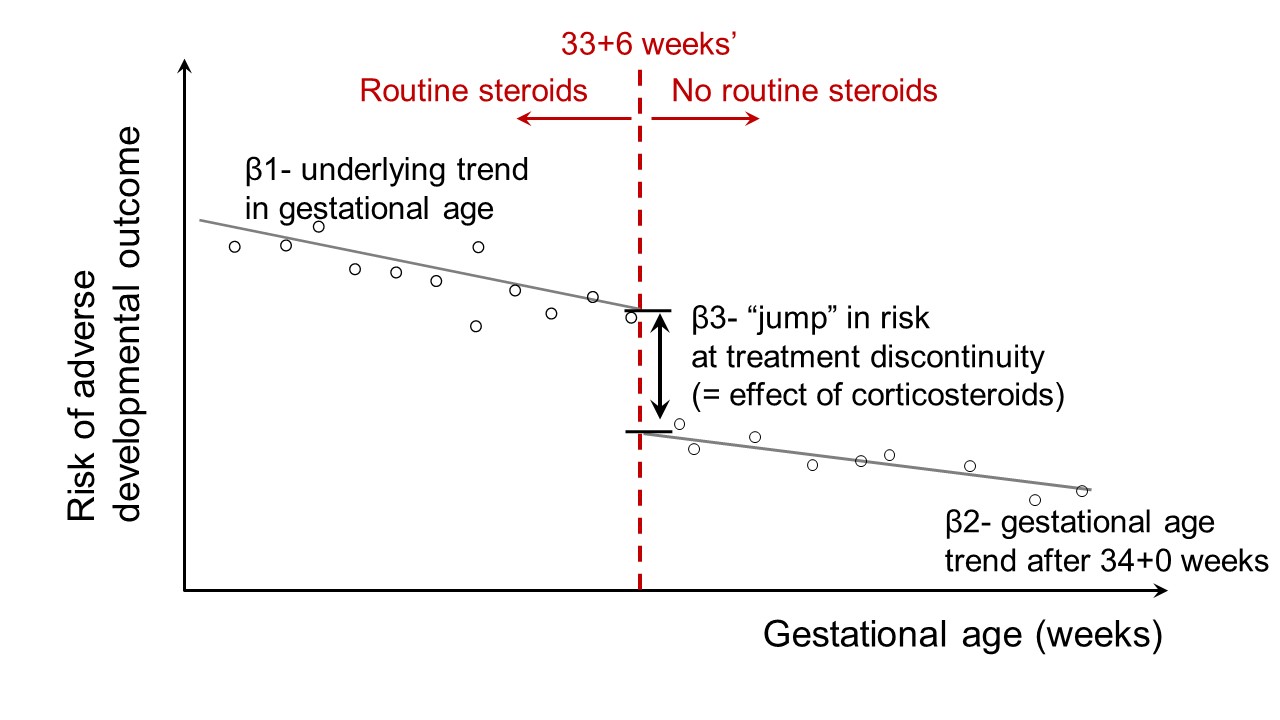

Supplement: S1 Fig — (DOCX) [file pmed.1003435.s001.docx]
