# Supplementary material for: Antenatal corticosteroid administration and early school age child development: A regression discontinuity study in British Columbia, Canada
Source: PLoS Med. 2020 Dec 7;17(12):e1003435. doi: 10.1371/journal.pmed.1003435 (PMC7721186; doi:10.1371/journal.pmed.1003435)
Supplement: S2 Fig — The vertical dashed line indicates the upper gestational age cut-off for routine administration of antenatal corticosteroids (33+6 weeks of gestation). The terms “β1” and “β2” estimate the underlying trend in risk of adverse neurodevelopmental outcomes associated with gestational age (as risks of most adverse outcomes decrease with advancing gestational age) before and after the cut-off, respectively. The term “β3” is the primary estimate of interest, which estimates if there is a “jump” or level change in risk at the gestational age at which corticosteroids stop being administered routinely (i.e., at the point of discontinuity in corticosteroid treatment practices). If antenatal corticosteroid administration causes adverse neurodevelopmental outcomes, we would expect a decrease in risk at 34+0 weeks, reflecting that risks dropped once fetuses were no longer exposed to antenatal corticosteroids. (DOCX) [file pmed.1003435.s002.docx]

**S2 Fig** Flow of participants

**526,525**

Live-born singleton births

British Columbia (2000-2013)

**294,807**

Day-specific ultrasound-confirmed estimate of gestational age available

No day-specific, ultrasound-confirmed estimate of gestational age

**143,860** (27.3%) LMP, no ultrasound

**46,140** (8.8%) No LMP, ultrasound in weeks only

**41,718** (7.9%) No LMP or ultrasound

**12,499** (4%) Left the province before age 4

**282,308**

Residing in British Columbia at age 4

**15,741**

Admitted for delivery admission 31+0 to 36+6 weeks’ gestation

**266,567** (94%) <31+0 or ≥37+0 weeks’ gestation

**5,562**

Early school age Early Development Index scores cohort

**10,176** (65%) Child’s school participated in testing a different year of 3-year testing cycle
