# Supplementary material for: Antenatal corticosteroid administration and early school age child development: A regression discontinuity study in British Columbia, Canada
Source: PLoS Med. 2020 Dec 7;17(12):e1003435. doi: 10.1371/journal.pmed.1003435 (PMC7721186; doi:10.1371/journal.pmed.1003435)
Supplement: S3 Fig — Vertical dashed line indicates the upper limited of recommended administration, 33+6 weeks (237 days). Circles indicate observed day-specific risks of neonatal respiratory morbidity/mortality; solid circles highlight the gestational ages coinciding with reduced antenatal corticosteroid administration. Solid line indicates the smoothed estimate of risk with 95% confidence interval (shaded area). (DOCX) [file pmed.1003435.s003.docx]

**S3 Fig** Neonatal respiratory morbidity or mortality among 15,741 births in British Columbia, Canada, 2000-2013. Vertical dashed line indicates the upper limited of recommended administration, 33+6 weeks’ (237 days). Circles indicate observed day-specific risks of neonatal respiratory morbidity/mortality; solid circles highlight the gestational ages coinciding with reduced antenatal corticosteroid administration. Solid line indicates the smoothed estimate of risk with 95% confidence interval (shaded area).

Routine steroids

No routine steroids
