# Supplementary material for: Antenatal corticosteroid administration and early school age child development: A regression discontinuity study in British Columbia, Canada
Source: PLoS Med. 2020 Dec 7;17(12):e1003435. doi: 10.1371/journal.pmed.1003435 (PMC7721186; doi:10.1371/journal.pmed.1003435)
Supplement: S4 Fig — Early Development Instrument (EDI) test results among 5,562 kindergarten-aged children in British Columbia, Canada, admitted for the delivery admission between 31+0 and 36+6 weeks of gestation, 2000 to 2013, for EDI subdomains of (a) communication skills, (b) emotional maturity, (c) language and cognitive development, (d) physical health and well-being, and (e) social competence. Vertical dashed line indicates the upper limited of recommended administration, 33+6 weeks (237 days). (DOCX) [file pmed.1003435.s004.docx]

**S4 Fig** Early Development Index (EDI) test results among 5562 kindergarten-aged children in British Columbia, Canada, admitted for the delivery admission between 31+0 and 36+6 weeks’ gestation, 2000-2013, for EDI subdomains of a) communication skills, b) emotional maturity, c) language and cognitive development, d) physical health & well-being, and e) social competence. Vertical dashed line indicates the upper limited of recommended administration, 33+6 weeks’ (237 days).
