# Supplementary material for: Antenatal corticosteroid administration and early school age child development: A regression discontinuity study in British Columbia, Canada
Source: PLoS Med. 2020 Dec 7;17(12):e1003435. doi: 10.1371/journal.pmed.1003435 (PMC7721186; doi:10.1371/journal.pmed.1003435)
Supplement: S5 Fig — (DOCX) [file pmed.1003435.s005.docx]

**S5 Fig** Distribution of select maternal-fetal characteristics across gestational age to verify the assumption that variables are continuous across the 34+0 weeks’ discontinuity in antenatal corticosteroid recommendations.

**S5 Fig con’t.** Distribution of select maternal-fetal characteristics across gestational age to verify the assumption that variables are continuous across the 34+0 weeks’ discontinuity in antenatal corticosteroid recommendations.
