# Supplementary material for: Antenatal corticosteroid administration and early school age child development: A regression discontinuity study in British Columbia, Canada
Source: PLoS Med. 2020 Dec 7;17(12):e1003435. doi: 10.1371/journal.pmed.1003435 (PMC7721186; doi:10.1371/journal.pmed.1003435)
Supplement: S1 Table — (DOCX) [file pmed.1003435.s006.docx]

**S1 Table.** Characteristics of pregnancies excluded due to lack of day-specific, ultrasound-based gestational age data among singleton births in British Columbia, Canada, April 1 2000-March 31 2013.

| **Maternal-fetal characteristic** | **Included cohort (n=294,807)**  mean ± standard deviation or n(%) | **Excluded cohort (n=231,718)**  mean ± standard deviation or n(%) |
| --- | --- | --- |
| Maternal age (years) | 31± 5 | 30± 6 |
| Nulliparity | 139,056 (47) | 102,932 (44) |
| Smoking in pregnancy | 24,960 (8) | 28,014 (13) |
| Pre-pregnancy body mass index (kg/m^2^)  Available body mass index | 24± 5  219,567 (74) | 24± 5  150,189 (65) |
| Hypertensive disorder of pregnancy | 17,308 (6) | 12,970 (6) |
| Diabetes in pregnancy | 32,011 (11) | 24099 (10) |
| Cesarean delivery | 88,083 (30) | 64,020 (28) |
| Labour induction | 62,930 (21) | 219,567 (20) |
| Delivered post-April 1 2008 | 151,812 (52) | 60,958 (26) |
|  |  |  |
| Male fetus | 150,547 (51) | 119,835 (52) |
| Birthweight | 3438± 538 | 3426± 560 |
| Gestational age at delivery in weeks  Preterm birth <37 weeks | 39± 2  21,641 (7) | 39± 2  17,989 (8) |
| 5-minute Apgar score <7 | 9621 (3) | 7604 (3) |
| Neonatal respiratory morbidity or mortality | 14891 (5) | 11,799 (5) |
