# Supplementary material for: Antenatal corticosteroid administration and early school age child development: A regression discontinuity study in British Columbia, Canada
Source: PLoS Med. 2020 Dec 7;17(12):e1003435. doi: 10.1371/journal.pmed.1003435 (PMC7721186; doi:10.1371/journal.pmed.1003435)
Supplement: S2 Table — (DOCX) [file pmed.1003435.s007.docx]

**S2 Table.** Effect of changing the gestational age window around the 34+0 week discontinuity when estimating the effect of antenatal corticosteroid administration practices among 5562 children in British Columbia, Canada, 2000-2013.

|  | **Estimated effect of corticosteroid administration practice (<34 weeks’ gestation vs. reference of ≥34 weeks)**  **Absolute difference in median scores^1^ [95% Confidence Interval]** | | | |
| --- | --- | --- | --- | --- |
| **Outcome** | **21-day window**  n=5,562 | **14-day window**  n=2,662 | **7-day window**  n=1,068 | **Model-selected optimal window^2^** |
| Total Early Development Index score (/50) | -0.5 [-2.2, 1.7] | -0.1 [-2.3, 2.3] | 1.8 [-1.8, 4.6] | -0.4 [-2.8, 2.0]  (8.7 days) |
| Communication skills score (/10) | -0.4 [-1.3, 0.8] | -0.6 [-1.3, 1.2] | -0.5 [-1.7, 1.7] | -0.2 [-1.3, 0.9]  (5.2 days) |
| Emotional maturity score (/10) | -0.2 [-0.6, 0.2] | -0.1 [-0.5, 0.3] | 0.4 [-0.2, 1.3] | 0.0 [-0.6, 0.5]  (6.7 days) |
| Language and cognitive development score (/10) | 0.5 [-0.3, 0.6] | 0.5 [-0.4, 0.7] | 0.4 [-0.4, 1.0] | 0.0 [-0.8, 0.7]  (7.0 days) |
| Physical health & well-being score (/10) | 0.0 [-0.2, 0.1] | 0 [-0.2, 0.1] | 0 [-0.5,0.3] | 0.0 [-0.8, 0.7]  (5.8 days) |
| Social competence score (/10) | -0.2 [-0.7, 0.4] | -0.1 [-0.7, 0.5] | 0.4 [-0.3, 1.1] | -0.2 [-0.8, 0.4]  (7.3 days) |
|  | **Excess cases per 100 births [95% Confidence Interval]** | | | |
| Developmentally vulnerable | 3.9 [-2.2, 10.0] | 5.6 [-0.2, 14.7] | 6.1 [-6.5, 19.5] | -0.4 [-15.4, 13.9]  (6.1 days) |
| Special needs designation | -0.5 [-4.2, 3.1] | -1.1 [-6.0, 4.4] | 0.1 [-6.7, 10.3] | -1.8 [-11.4, 6.9]  (7.6 days) |
| 1 Coefficients estimate effects among infants routinely exposed to antenatal corticosteroids (i.e., admitted <34+0 weeks) compared with those unexposed | | | | |
| 2 Estimates mean rather than median. Optimal window (i.e., bandwidth) selected using the default ‘mserd’ option in the Stata 16 command rdrobust, which uses one common mean squared error (MSE)-optimal bandwidth selector for the regression discontinuity treatment-effect estimator. | | | | |
