# Supplementary material for: Antenatal corticosteroid administration and early school age child development: A regression discontinuity study in British Columbia, Canada
Source: PLoS Med. 2020 Dec 7;17(12):e1003435. doi: 10.1371/journal.pmed.1003435 (PMC7721186; doi:10.1371/journal.pmed.1003435)
Supplement: S3 Table — (DOCX) [file pmed.1003435.s008.docx]

| **Outcome** | **Estimated effect of corticosteroid administration practice (<34 weeks’ gestation vs. reference of ≥34 weeks)** | |
| --- | --- | --- |
|  | **Absolute difference in median scores [95% confidence intervaI]** |  |
| Total Early Development Index score (/50) | 2.1 [-1.5, 4.0] | - |
| Communication skills score (/10) | 0.2 [-1.3,2.0] | - |
| Emotional maturity score (/10) | 0.0 [-0.6, 0.7] | - |
| Language and cognitive development score (/10) | 0.6 [0.4, 1.0] | - |
| Physical health & well-being score (/10) | 0.0 [-0.4, 0.5] | - |
| Social competence score (/10) | 0.3 [-0.4, 1.0] | - |
|  |  |  |
|  | **Excess cases per 100 births [95% confidence interval]** | **Risk ratio [95% confidence interval]** |
| Developmentally vulnerable | 0.9 [-8.7,11.5] | 1.0 [0.7, 1.4] |
| Special needs designation | 0.4 [-5.7,7.4] | 1.0 [0.5, 1.8] |

**S3 Table.** Effect of restricting to births admitted after April 1, 2008 when estimating the effect of antenatal corticosteroid administration practices among 5562 children in British Columbia, Canada.
