# Supplementary material for: Antenatal corticosteroid administration and early school age child development: A regression discontinuity study in British Columbia, Canada
Source: PLoS Med. 2020 Dec 7;17(12):e1003435. doi: 10.1371/journal.pmed.1003435 (PMC7721186; doi:10.1371/journal.pmed.1003435)
Supplement: S4 Table — (DOCX) [file pmed.1003435.s009.docx]

**S4 Table**. Characteristics of children with *vs.* without missing Total Early Development Index (EDI) scores in British Columbia, Canada, 2000-2013.

| **Maternal-fetal characteristic** | **Missing EDI data**  mean ± standard deviation or n(%) | **Available EDI data**  mean ± standard deviation or n(%) |
| --- | --- | --- |
| n | 50 | 5512 |
| Maternal age (years) | 29± 5 | 31± 6 |
| Nulliparity | 20 (40) | 2753 (50) |
| Smoking in pregnancy | 8 (16) | 706 (13) |
| Pre-pregnancy Body Mass Index (kg/m^2^)^1^ | 24± 5 | 25± 5 |
| Hypertensive disorder of pregnancy^2^ | <10 (-) | 861 (16) |
| Diabetes in pregnancy | 13 (26) | 904 (16) |
| Cesarean delivery | 19 (38) | 2059 (37) |
| Labour induction | 12 (24) | 1407 (26) |
|  |  |  |
| Male fetus | 32 (64) | 3131 (57) |
| Birthweight | 2671± 567 | 2669± 536 |
| Gestational week at admission for delivery^2^  31  32  33  34  35  36 | <10 (-)  6 (12)  <10 (-)  <10 (-)  11 (22)  26 (52) | 196 (4)  297 (5)  401 (7)  662 (12)  1280 (23)  2676 (49) |
| 5-minute Apgar score <7^2^ | <10 (-) | 494 (9) |
| Neonatal respiratory morbidity or mortality^2^ | <10 (-) | 1151 (21) |
| ^1^Among 3932 and 36 women with and without available EDI data with available BMI  ^2^Exact figure not presented due to restrictions on presenting small cell sizes | | |
