# Supplementary material for: Antenatal corticosteroid administration and early school age child development: A regression discontinuity study in British Columbia, Canada
Source: PLoS Med. 2020 Dec 7;17(12):e1003435. doi: 10.1371/journal.pmed.1003435 (PMC7721186; doi:10.1371/journal.pmed.1003435)
Supplement: S1 Protocol — (PDF) [file pmed.1003435.s010.pdf]

Pre-analysis plan for "Short term benefits but long term harm? Assessing the consequences of antenatal corticosteroid administration for child neurodevelopment"

Jennifer A Hutcheon[1], Sam Harper[2], Amanda Skoll[1], Myriam Srour[3], Jessica Liauw[1], Erin Strumpf[2,4]

[1]Department of Obstetrics & Gynaecology, University of British Columbia
[2] Department of Epidemiology, Biostatistics, and Occupational Health, McGill University
[3] Department of Pediatric Neurology, McGill University
[4] Department of Economics, McGill University

Purpose

This document describes a pre-analysis plan for a study examining the child health consequences of antenatal corticosteroid administration in a population-based cohort of linked administrative and clinical records from British Columbia, Canada. We use a regression discontinuity design that exploits the pronounced change in antenatal corticosteroid administration practices based on a clinical practice guideline that recommended administration up to 33 weeks, 6 days of gestation (33+6 weeks), but not at or beyond 34+0 weeks. This pre-analysis plan was written after the individual datasets had been received and some descriptive statistics calculated for key variables, but prior to the linkage of the datasets or analyses linking exposure with longer-term child health outcomes.

Introduction

Randomized trials have consistently shown that administering antenatal corticosteroids to pregnant women at risk of preterm labour reduces neonatal mortality and respiratory morbidity.[1] As a result, Canadian and international practice guidelines unequivocally advise that all eligible women at 26 to 34 weeks' gestation at risk of an imminent preterm delivery (due to spontaneous onset of labour, preterm premature rupture of membranes, or pregnancy complication indicating an early delivery) should be given antenatal steroids.[2,3] Antenatal steroids are a *WHO Priority Life-Saving Medicine*, and administration rates are audited as an indicator of perinatal care quality in multiple jurisdictions including Canada.

However, we still lack high quality evidence on the longer-term safety of antenatal corticosteroids. Select studies have signalled that antenatal steroids may have harmful consequences for child neurodevelopment,[4] and obstetrical leaders are beginning to advocate for more restricted use until long-term safety has been convincingly demonstrated.[5-7] However, generating rigorous evidence on the effects of antenatal steroids for child neurodevelopment is challenging. Follow-up studies of randomized trials were hampered by low follow-up rates and small sample sizes,[8-11] and observational studies are highly susceptible to confounding.[12] Without high-quality safety evidence, the unproven potential for harm may be causing a treatment with proven neonatal benefits to be withheld unnecessarily.

When randomization is infeasible, regression discontinuity (RD) studies can provide credible causal effect estimates.[13] The design exploits differences in care between individuals immediately on either side of a clinical cut-point as a quasi-randomization tool. With antenatal corticosteroids guidelines recommend administration of corticosteroids up to 33+ 6 weeks' gestation – but not one day later, at 34 weeks+ 0 days, so infants born as little as hours apart have substantially different chances of receiving the treatment. We propose to use this novel design to generate rigorous evidence on the longer-term safety of antenatal steroids. We also propose to examine if antenatal corticosteroid administration's protective effect on respiratory morbidity extends into early childhood.

Objectives

To determine if antenatal corticosteroid administration:

1) decrease <u>kindergarten child development scores</u> (primary outcome)
2) increase risks of adverse neurodevelopmental outcomes such <u>cerebral palsy</u> or <u>attention deficit hyperactivity disorder (ADHD) (secondary outcomes)</u>, and
3) decrease the risk of <u>lower respiratory tract infection hospital admission under the age of two</u> or <u>childhood asthma (secondary outcomes).</u>

Overview of research plan

We will estimate the effect of antenatal corticosteroid use on child neurodevelopmental outcomes using a novel quasi-experimental design that exploits the clinical cut-off of antenatal corticosteroids administration at 34 weeks. We will link abstracted obstetrical and neonatal chart records from approximately 16,000 preterm births delivered from 2000 to 2013 contained in the British Columbia (BC) Perinatal Data Registry with longer-term child health information contained in BC population-based administrative databases and kindergarten testing data. We will isolate the difference in test scores and adverse child health outcomes occurring at 34 weeks using a regression discontinuity design, while accounting for the general decrease in adverse outcomes associated with advancing gestational age. The study was approved by the Research Ethics Board of the BC Children's & Women's Hospital/University of British Columbia (H18-00620).

Study population

Our study population will be drawn from all singleton deliveries in British Columbia, Canada admitted

for the delivery admission between 31+0 and 36+6 weeks, inclusive, from April 1, 2000 to March 31, 2013. Late pregnancy terminations and pregnancies with no ultrasound-confirmed, day-specific estimate of gestational age will be excluded.

Data

Our study cohort will be created through a linkage of British Columbia's comprehensive population-based databases of detailed obstetrical medical records, medication prescriptions, physician billing, hospital discharge records, vital statistics, and kindergarten child development assessments. The linkage will be performed by PopData BC, a multi-university organization that facilitates the use of the province's databases for research purposes. Maternal and infant personal health numbers and dates of birth will be used as unique identifiers. The linked data are accessed in anonymized format on a secure server to ensure data confidentiality and security. Follow-up data up to March 31, 2018 will be included, ensuring a minimum of 5 years follow-up for all children.

BC Perinatal Data Registry: The BC Perinatal Data Registry contains abstracted obstetrical and neonatal medical records for >99% of deliveries in the province since April 1, 2000. Data quality are maintained through the use of provincially-standardized medical record forms, standardized training of abstractors (certified following a 2-year training program), and regular data checks for plausibility and missing values. Validation studies have established the accuracy and completeness of the database.[14]

Early childhood development data: British Columbia has been conducting standardized assessments of child development at kindergarten age since 2001 using the Early Development Instrument, a questionnaire used across Canada and internationally.[15] Data are collected in 3-year waves, so are only available for approximately one-third of children in the province a given year.

Hospital Discharge Abstract Database: The BC Hospital Discharge Abstract database contains International Classification of Disease (ICD) codes summarizing the diagnoses and procedures for all discharges, transfers and deaths of inpatients in acute care hospitals in British Columbia. We will only use the first 16 diagnostic coding spots, to ensure that the number of diagnostic coding spots is consistent throughout the study period (a greater number of spots are available in more recent years).

Physician billing records: These records contain data on medically required services provided by fee-for-service practitioners to individuals covered by BC's universal insurance program. These are collected in ICD-9 format. These codes have been validated at the population level.[16]

BC Vital Statistics Deaths: The BC Vital statistics Agency records all deaths registered in BC.

Pharmanet: The BC PharmaNet database documents all prescription medications dispensed by community pharmacies in the province, as well as all prescriptions dispensed from hospital outpatient pharmacies for patient use at home. Data are available from Jan 1, 1996 onwards.

Consolidation file: The Consolidation file is a central demographic file containing information such as location of residence, enabling linkage of an individual's postal code of residence with postal code level census information such as median neighbourhood income quintile.

Exposure measurements

Gestational age in days will be calculated using the gestational age algorithm used clinically during the study period. The estimate of gestational age will be based on the last menstrual period if this estimate is similar to the estimate based on early ultrasound (≤5 days if the ultrasound was performed <14 weeks; ≤10 days if the ultrasound was performed <20 weeks). If the gestational age estimate based on last menstrual period disagrees with early ultrasound, or no last menstrual period estimate is available, the ultrasound estimate will be used. Day-specific estimates of ultrasound-based gestational age are available in the BC Perinatal Data Registry as of April 1, 2008. Prior to this period, we will calculate day-specific gestational age based on the date of the last menstrual period among women

whose ultrasound-based estimate of gestational age (in weeks) is the same or within 1 week of their estimate based on last menstrual period (in weeks). We will use gestational age in days at the time of maternal admission for the delivery as our unit of analysis, which better approximates timing of antenatal corticosteroid administration compared with gestational age at delivery. We will conduct sensitivity analyses to explore the potential for bias due to exclusion of women with missing day-specific gestational age estimates.

Antenatal corticosteroid administration is available in the BC Perinatal Data Registry as a binary variable indicating the administration of corticosteroids during the delivery admission for the purpose of fetal lung maturation. This can be any type of corticosteroid (e.g., betamethasone or dexamethasone) and does not reflect dosing information. This variable is known to underestimate the true administration rates, as administration during an antenatal admission shortly prior to delivery is not captured (i.e., a patient is admitted, receives antenatal corticosteroids, but is discharged without delivering). We will obtain this variable to confirm the change in the probability of receiving antenatal corticosteroids according to gestational age, but are using the gestational age discontinuity, not the provision of steroids *per se*, as our primary exposure.

Child health outcomes

We will examine adverse outcomes from different points along the spectrum of potential neurodevelopmental consequence, from more subtle differences in lower school development scores to serious adverse neurodevelopmental outcomes such as cerebral palsy. We will also determine if antenatal corticosteroid administration is protective against childhood respiratory conditions.

Our **primary outcome** will be the standardized domain scores of the Early Development Instrument (EDI). The Early Development Instrument was developed by researchers at McMaster University to measure children's development in kindergarten.[15] The EDI provides a holistic assessment of children's development through 104 questions across five domains: physical health and well-being, social competence, emotional maturity, language and cognitive development, and communication skills. The assessments are completed by kindergarten teachers in February for each child in their class, after the teachers have had several months of interaction with the children**.**

The EDI has been shown to be a reliable and psychometrically-sound tool to measure population-level child development, and is a good predictor of adult health, education and social outcomes.[17] It is used by governments in multiple Canadian provinces (such as Ontario, Manitoba, and British Columbia) and internationally (Australia, Hong Kong) to assess kindergarten readiness and child development. Its widespread use will facilitate the interpretation and comparison of scores from our research.

Each of the five EDI domains are relevant to the detection of adverse neurodevelopmental outcomes. For example, in the physical health domain, questions such as "Can the child manipulate objects?" will capture concerns about fine motor skills and coordination, which are more subtle outcomes on the spectrum to cerebral palsy. Likewise, in the emotional maturity domain, questions such as "Is the child distractable, has trouble sticking to any activity?" assesses characteristics of ADHD. The scores from each domain are standardized into mean scores, and will be examined as continuous variables. We will consider a difference in the total EDI of 5 or greater to be clinical significant. This would reflect a child who scoring 1 point out of 10 lower on each of the 5 subscale domains, and corresponds approximately to the difference between the score of a child on the 50th percentile (41/50) and that of a child on the 25th percentile score (35/50). We will also examine a composite outcome of 'developmentally vulnerable', which is defined as a score <10th percentile on at least two domains (secondary outcome), and an indication of whether the child was identified as having special needs.[15]

Other **secondary neurodevelopmental outcomes** will be cerebral palsy and ADHD, based on previously-published definitions from BC administrative data.[18-20] Cerebral palsy will be identified using

International Classification of Disease (ICD) codes from hospitalization and physician billing records.[20] ADHD will be identified using prescription records for methylphenidate (Ritalin) and other ADHD medications, based on pediatric ADHD prescribing patterns in Canada described in recent national Canadian Primary Care Sentinel Surveillance Network data.[18,19,21]. Our **secondary respiratory outcomes** will be a diagnosis of asthma (based on two or more physician billing records or a hospitalization for asthma) and any hospital admission for lower respiratory tract infection during the first two years of life. See Table 1 for detailed outcome definitions.

For all analyses, infant or child deaths (identified based on vital statistics records) will be assigned the lowest observed score (for EDI scores) or occurrence of the adverse outcome. This will be done to prevent selection bias introduced from differential losses to follow up according to antenatal corticosteroid administration status (i.e., having fewer cases of cerebral palsy in the untreated group because those infants who didn't receive treatment didn't survive infancy to develop the condition).[22]

Table 1. Study outcome definitions

Outcome	Data source	Definition
Kindergarten age child development scores	Early Development Instrument	Mean domains scores for each of:<br>1. Language and cognitive development<br>2. Physical well-being<br>3. Social competence<br>4. Emotional maturity<br>5. Communication and general knowledge
Developmentally vulnerable	Early Development Instrument	Score <10th percentile for two or more domains[15]
Special needs	Early Development Instrument	Flag indicating the child has special needs
Cerebral palsy	Hospital discharge records and physician billing claims	ICD-9 code 343 or ICD-10 code G80, with two confirmatory diagnoses if initial diagnosis was prior to age 3[20]
ADHD	PharmaNet	≥ 1 prescription of methylphenidate, amphetamine and its derivatives, or atomoxetine (ATC codes N06B)[18,19,21]
Childhood asthma	Hospital discharge records and physician billing claims	ICD-9 code 493 or ICD-10 code J45, with two confirmatory diagnoses if based on physician billing, or diagnosis in the primary diagnosis position (most responsible) for hospital admissions.
Hospital admission for lower respiratory tract infection in the first two years of life	Hospital discharge records	Hospitalization of a child aged 2 or less with any of the following ICD-9 or ICD-10 codes:<br>466.0 or J20 Acute bronchitis<br>466.1 or J21 Acute bronchiolitis<br>480–486 or J12-J18 Pneumonia<br>487 or J10- J11 Influenza, excluding cases due to certain identified influenza viruses

Statistical analysis

We will isolate the effects of antenatal corticosteroid administration practices using a regression discontinuity design. This design exploits the fact that an infant's probability of receiving antenatal corticosteroids changes significantly based on whether its mother presented to hospital before or after

the 34 week gestational age cut-off. The design assumes that infants immediately below the 34-week threshold are exchangeable with those immediately above the threshold except for the probability of receiving antenatal steroids, mimicking random treatment assignment. In the absence of any treatment effect, outcome rates should be smooth across the 34-week threshold, therefore any estimated change in the outcome at 34 weeks can be assumed to be due to antenatal corticosteroid treatment.

We will implement the design using a multivariable quantile regression model estimating the 50th percentile (for our primary outcome of EDI scores), multivariable log-binomial models (for binary outcomes such as developmentally vulnerable, special needs, and lower respiratory tract infection hospitalization in the first two years of life), and pooled logistic regression models (for binary outcomes where children born in different study years have follow-up time such as ADHD and asthma). In the event that our log-binomial models fail to converge, we will use logistic regression and convert odds ratios to risk ratios using postestimation commands.

Our primary model will include delivery admissions between 31+0 and 36+6 weeks. We will weight observations according to their proximity to the cut-off of 34+0 using a triangular kernel, such that observations closest to the discontinuity are assigned greater weight and those furthest from the discontinuity are given less weight. This weighting is particularly important in this cohort as the probability of birth increases with advancing gestational age, so the largest number of observations will occur furthest from the discontinuity (i.e., at 36+6 weeks).

We will use standard regression discontinuity methods to estimate the size of the discontinuity in child health outcomes due to crossing the threshold at 34+0 weeks (Lee & Lemieux 2010). We will estimate the following regression models for individuals in our sample using a 3-week bandwidth around the gestational age threshold:

$$Y_i = \alpha_0 + \alpha_1 T_i + \alpha_2 T_i * (GA - T) + \alpha_3 (1-T_i) * (GA - T) + \delta X_i + e_i \quad (3)$$

where Y_i is an outcome for child *i* and T is an 0/1 indicator for being below the threshold at 34+0 weeks. We include separate trends for modeling gestational age (*GA*) above ($\alpha_1 + \alpha_3$) and below (α_1) the threshold, and a vector for any other demographic or clinical covariates (X_i). As is good practice for regression discontinuity designs, we will also evaluate models with squared terms for the underlying association with gestational age in some specifications, with the final specification based on the Akaike Information Criterion (AIC). We will bootstrap 95% confidence intervals.

The coefficient T estimates the intention-to-treat (ITT) effect of the intervention, reflecting the impact of the guideline in a real-world setting (in which imperfect compliance with corticosteroid administration occurs because of incomplete courses, mis-timed administration, and insufficient time to administer corticosteroids prior to delivery). We will interpret this coefficient as our primary estimate of the effect of antenatal corticosteroids administration practices on each child health outcome. We will estimate a "per protocol" effect of antenatal steroids by dividing the discontinuity in outcomes estimated by our model by the magnitude of the discontinuity in corticosteroid administration at 34 weeks (estimated in a separate model), but will interpret results cautiously because of the known under-documentation of antenatal corticosteroid administration in our Perinatal Database variable. This under-documentation would likely underestimate the magnitude of the discontinuity in corticosteroid administration at 34 weeks, which, in turn, would over-estimate the causal effect of corticosteroid administration on child health outcomes.

Sensitivity analyses

We have pre-specified a number of sensitivity analyses to assess the robustness of our findings:

<u>Alternative outcome definitions</u>

1. Hospitalization for lower respiratory tract infection in the first two years of age:
    a) We will model the total number of hospitalizations per child in the first two years after birth rather than the occurrence of any hospitalization. We have opted to use 'any hospitalization' as our primary outcome as it is possible that some subsequent hospitalizations are re-admissions for the same infection rather than new infections.
    b) We will examine the outcome of RSV bronchiolitis rather than the broader outcome of lower respiratory tract infection (ICD-9 code 466.11; ICD-10 code J21.0).
    c) We will only include lower respiratory tract infections during the typical RSV season (November to April)
2. Asthma:
    a) We will examine hospitalization for asthma alone as an outcome (as opposed to a diagnosis using either hospitalization or physician visits)
    b) We will identify asthma hospitalization in any position of the hospital discharge summary (rather than only in the primary position)
3. Cerebral palsy:
    a) We will restrict cases of cerebral palsy to those that also have a code for complications including: feeding difficulty/dysphagia; dystonia; spasticity; or developmental delay
    b) We will restrict analyses to births admitted to hospital prior to May 2011, when a Society of Obstetricians and Gynaecologists of Canada guideline was issued recommending the administration of Magnesium Sulphate for fetal neuroprotection for imminent preterm birth. The guideline recommended administration up to 31+6 weeks, but provided institutional discretion to adopt higher gestational age thresholds (<34 weeks).
4. ADHD:
    a) We will use define ADHD using physician billing records in addition to N06B prescriptions (ICD-9 code 314.0).

<u>Excluding women who may have received corticosteroids in a prior admission</u>

To account for potential under-reporting of antenatal corticosteroids caused by administration during an antenatal hospital admission that did not result in delivery (which is not documented in the BC Perinatal Database), we will conduct an analysis restricted to women with no prior hospitalizations during the pregnancy. We will derive our primary estimates of the effects of corticosteroids (i.e., "per protocol" analysis) in this subgroup.

<u>Assessing comparability of groups</u>

We will assess comparability around the 34+0 week cut-off by plotting maternal-fetal characteristics according to gestational age. Specifically, we will examine maternal age, parity, pre-pregnancy BMI, smoking in pregnancy, neighbourhood income quintile, fetal sex, diabetes status, pre-eclampsia status, mode of delivery (Cesarean vs vaginal), type of labour onset (spontaneous vs iatrogenic vs none), placental abruption, and fever in labour/chorioamnionitis. We have no reason to believe these factors will change discontinuously at 34 weeks, but will control for them in our models in the event of meaningful differences.

<u>Alternative gestational age windows</u>

Our primary analysis will include all infants whose mothers were admitted to hospital for the delivery admission between 31+0 and 36+6 weeks, inclusive. However, we will confirm that the point estimate from our primary analysis is similar to that obtained if the gestational age window is restricted to 32+0 to 35+6 weeks, inclusive. We will also repeat analyses restricting the gestational age window to 33+0 to 34+6 weeks, however, we anticipate that estimates will be less stable in this gestational age window given the relatively small sample size.

REFERENCES

1. Roberts D, Brown J, Medley N, Dalziel SR. Antenatal corticosteroids for accelerating fetal lung maturation for women at risk of preterm birth. The Cochrane database of systematic reviews 2017;3:CD004454.
2. Royal College of Obstetricians and Gynaecologists. Antenatal Corticosteroids to Reduce Neonatal Morbidity and Mortality. London, UK; 2010.
3. Society of Obstetricians and Gynaecologists of Canada Expert Opinion. Antenatal corticosteroid therapy for fetal lung maturation. Journal of Obstetrics and Gynaecology Canada 2003;25:45-8.
4. Waffarn F, Davis EP. Effects of antenatal corticosteroids on the hypothalamic-pituitary-adrenocortical axis of the fetus and newborn: experimental findings and clinical considerations. American Journal of Obstetrics and Gynecology 2012;207:446-54.
5. Aiken CE, Fowden AL, Smith GC. Antenatal glucocorticoids prior to cesarean delivery at term. JAMA Pediatrics 2014;168:507-8.
6. Crowther CA, Harding JE. Antenatal Glucocorticoids for Late Preterm Birth? The New England Journal of Medicine 2016;374:1376-7.
7. Nowik CM, Davies GA, Smith GN. We Should Proceed With Caution When It Comes to Antenatal Corticosteroids After 34 Weeks. Journal of Obstetrics and Gynaecology Canada 2017;39:49-51.
8. Dalziel SR, Lim VK, Lambert A, et al. Antenatal exposure to betamethasone: psychological functioning and health related quality of life 31 years after inclusion in randomised controlled trial. BMJ 2005;331:665.
9. Dessens AB, Haas HS, Koppe JG. Twenty-year follow-up of antenatal corticosteroid treatment. Pediatrics 2000;105:E77.
10. Salokorpi T, Sajaniemi N, Hallback H, Kari A, Rita H, von Wendt L. Randomized study of the effect of antenatal dexamethasone on growth and development of premature children at the corrected age of 2 years. Acta Paediatrica 1997;86:294-8.
11. Stutchfield PR, Whitaker R, Gliddon AE, Hobson L, Kotecha S, Doull IJ. Behavioural, educational and respiratory outcomes of antenatal betamethasone for term caesarean section (ASTECS trial). Archives of Disease in Childhood Fetal and Neonatal Edition 2013;98:F195-200.
12. Sotiriadis A, Tsiami A, Papatheodorou S, Baschat AA, Sarafidis K, Makrydimas G. Neurodevelopmental Outcome After a Single Course of Antenatal Steroids in Children Born Preterm: A Systematic Review and Meta-analysis. Obstetrics and Gynecology 2015;125:1385-96.
13. Moscoe E, Bor J, Barnighausen T. Regression discontinuity designs are underutilized in medicine, epidemiology, and public health: a review of current and best practice. Journal of Clinical Epidemiology 2015;68:122-33.
14. Frosst G, Hutcheon J, Joseph KS, Kinniburgh B, Johnson C, Lee L. Validating the British Columbia Perinatal Data Registry: a chart re-abstraction study. BMC Pregnancy and Childbirth 2015;15:123.
15. Janus M, Offord D. Psychometric properties of the Early Development Instrument (EDI): a teacher-completed measure of children's readiness tolearn at school entry. Canadian Journal of Behavioural Science 2007;39:1-22.
16. Hu W. Diagnostic Codes in MSP Claim Data, Summary Report. Victoria, BC.; 1996.
17. Janus M, Harrison LJ, Goldfeld S, Guhn M. International research utilizing the Early Development Instrument (EDI) as a measure of early child development. Early Childhood Research Quarterly 2016;35:S1-5.
18. Brehaut JC, Miller A, Raina P, McGrail KM. Childhood behavior disorders and injuries among children and youth: a population-based study. Pediatrics 2003;111:262-9.

19. Miller AR, Brehaut JC, Raina P, McGrail KM, Armstrong RW. Use of medical services by methylphenidate-treated children in the general population. Ambulatory Pediatrics 2004;4:174-80.
20. Smith L, Kelly KD, Prkachin G, Voaklander DC. The prevalence of cerebral palsy in British Columbia, 1991-1995. The Canadian Journal of Neurological Sciences 2008;35:342-7.
21. Morkem R, Patten S, Queenan J, Barber D. Recent Trends in the Prescribing of ADHD Medications in Canadian Primary Care. Journal of attention disorders 2017:1087054717720719.
22. Rouse DJ, Hirtz DG, Thom E, et al. A randomized, controlled trial of magnesium sulfate for the prevention of cerebral palsy. The New England Journal of edicine 2008;359:895-905.
