# Supplementary material for: Antenatal corticosteroid administration and early school age child development: A regression discontinuity study in British Columbia, Canada
Source: PLoS Med. 2020 Dec 7;17(12):e1003435. doi: 10.1371/journal.pmed.1003435 (PMC7721186; doi:10.1371/journal.pmed.1003435)
Supplement: S1 Checklist — (PDF) [file pmed.1003435.s011.pdf]

STROBE Statement—Checklist of items that should be included in reports of ***cohort studies***

	Item No		Recommendation	
**Title and abstract**	1	✓	(*a*) Indicate the study's design with a commonly used term in the title or the abstract	
		✓	(*b*) Provide in the abstract an informative and balanced summary of what was done and what was found	
**Introduction**				
Background/rationale	2	✓	Explain the scientific background and rationale for the investigation being reported	Introduction
Objectives	3	✓	State specific objectives, including any prespecified hypotheses	Introduction, last paragraph
**Methods**				
Study design	4	✓	Present key elements of study design early in the paper	Introduction, 3rd paragraph
Setting	5	✓	Describe the setting, locations, and relevant dates, including periods of recruitment, exposure, follow-up, and data collection	Methods, paragraph 1 (study pop'n)
Participants	6	✓	(*a*) Give the eligibility criteria, and the sources and methods of selection of participants. Describe methods of follow-up	Methods, paragraph 1 (study pop'n)
			(*b*) For matched studies, give matching criteria and number of exposed and unexposed	n/a
Variables	7	✓	Clearly define all outcomes, exposures, predictors, potential confounders, and effect modifiers. Give diagnostic criteria, if applicable	Methods, paragraphs 2-8 (study variables)
Data sources/ measurement	8*	✓	For each variable of interest, give sources of data and details of methods of assessment (measurement). Describe comparability of assessment methods if there is more than one group	Methods, paragraphs 2-8 (study variables)
Bias	9	✓	Describe any efforts to address potential sources of bias	Methods, para 7,12
Study size	10	✓	Explain how the study size was arrived at	S1 Figure
Quantitative variables	11	✓	Explain how quantitative variables were handled in the analyses. If applicable, describe which groupings were chosen and why	Methods, para 12
Statistical methods	12	✓	(*a*) Describe all statistical methods, including those used to control for confounding	Methods, para 9-12
			(*b*) Describe any methods used to examine subgroups and interactions	n/a
		✓	(*c*) Explain how missing data were addressed	Methods, para 12
			(*d*) If applicable, explain how loss to follow-up was addressed	n/a
		✓	(*e*) Describe any sensitivity analyses	Methods, para 12
**Results**				
Participants	13*	✓	(a) Report numbers of individuals at each stage of study—eg numbers potentially eligible, examined for eligibility, confirmed eligible, included in the study, completing follow-up, and analysed	S1 Figure
		✓	(b) Give reasons for non-participation at each stage	S1 Figure
		✓	(c) Consider use of a flow diagram	S1 Figure
Descriptive data	14*	✓	(a) Give characteristics of study participants (eg demographic, clinical, social) and information on exposures and potential confounders	Table 1, S1 Table
		✓	(b) Indicate number of participants with missing data for each variable of interest	Figure S1, Table 2
			(c) Summarise follow-up time (eg, average and total amount)	n/a
Outcome data	15*	✓	Report numbers of outcome events or summary measures over time	
Main results	16	✓	(*a*) Give unadjusted estimates and, if applicable, confounder-adjusted estimates and their precision (eg, 95% confidence interval). Make clear which confounders were adjusted for and why they were included	Table 2
			(*b*) Report category boundaries when continuous variables were categorized	n/a
		✓	(*c*) If relevant, consider translating estimates of relative risk into absolute risk for a meaningful time period	Table 2

Other analyses	17 ✓	Report other analyses done—eg analyses of subgroups and interactions, and sensitivity analyses	Results, last paragraph
**Discussion**			
Key results	18 ✓	Summarise key results with reference to study objectives	Discussion, paragraph 1
Limitations	19 ✓	Discuss limitations of the study, taking into account sources of potential bias or imprecision. Discuss both direction and magnitude of any potential bias	Discussion, para 7-9
Interpretation	20 ✓	Give a cautious overall interpretation of results considering objectives, limitations, multiplicity of analyses, results from similar studies, and other relevant evidence	Discussion, para 2-5
Generalisability	21 ✓	Discuss the generalisability (external validity) of the study results	Discussion, para 9
**Other information**			
Funding	22 ✓	Give the source of funding and the role of the funders for the present study and, if applicable, for the original study on which the present article is based	Title page

*Give information separately for exposed and unexposed groups.

Note: An Explanation and Elaboration article discusses each checklist item and gives methodological background and published examples of transparent reporting. The STROBE checklist is best used in conjunction with this article (freely available on the Web sites of PLoS Medicine at http://www.plosmedicine.org/, Annals of Internal Medicine at http://www.annals.org/, and Epidemiology at http://www.epidem.com/). Information on the STROBE Initiative is available at http://www.strobe-statement.org.
