# Supplementary material for: Antenatal corticosteroid administration and early school age child development: A regression discontinuity study in British Columbia, Canada
Source: PLoS Med. 2020 Dec 7;17(12):e1003435. doi: 10.1371/journal.pmed.1003435 (PMC7721186; doi:10.1371/journal.pmed.1003435)
Supplement: Attachment — Submitted filename: hutcheon.pdf [file pmed.1003435.s012.pdf]

This paper reports an analysis of linked pregnancy and child development data–sets.

I was asked for a statistical report and I interpret that to include all aspects of the design and conduct of the study.

Points of detail

Page 6 I am afraid I did not grasp the issue of the 3–year waves. Does this mean that every third year all children in a three year age band are tested and then two years elapse before the next wave? That does not seem to fit with the rest of the text. Supplementary Figure 1 suggests something different too.

Page 8 Presumably the kernel is chosen to fall to zero at the limits of the data?

Page 8 Presumably a quadratic term for age is meant?

Page 8 The multiple imputation (MI) model is not really described here and could use a bit more detail about the variables in the model, which variables needed imputing, how many sets were generated and how much was missing for instance. This could go into the supplement if the authors feel it would break up the flow here.

Page 9, Figure 3 I am not sure why it is necessary to highlight the points near the discontinuity especially as that does not seem to have been done in Figure 4.

Page 10 The report of the MI here suggests that what happened was that there was item missing on the EDI and that was what was imputed. Is that the case?

Pages 12 and 13 I thought the strengths and limitations section was very fair and balanced.

Point of more substance

The authors present their results in the sequence: description, details of intervention, effect of morbidity and mortality, effect on development, sensitivity analyses. When I first read the results section and when I first looked at Figures 1 to 4 I almost missed the main point of the article, the point which

is summarised in the first sentence of the discussion. Is there some way of re–structuring this to make the main message more prominent? For instance the analysis of morbidity and mortality is more like a sensitivity analysis to confirm for us that the method would be capable of confirming a well known result. Figure 3 is much more prominent than Figure 4 as 4 contains small sub–figures, perhaps move 3 into 4 or make 4 three figures? I would also make Figure 1 more schematic as the unwary reader might think it shows actual results. I would label the y–axis 'Risk of event', replace the red text with 'Intervention', 'No intervention' and replace 'effect of corticosteroids' by 'effect of intervention'. I agree that the absence of axis tick marks rather gives the game away and when printed the caption will be with the figure not pages away.

Summary

Mostly minor points.

Michael Dewey
