# Supplementary material for: Antenatal corticosteroid administration and early school age child development: A regression discontinuity study in British Columbia, Canada
Source: PLoS Med. 2020 Dec 7;17(12):e1003435. doi: 10.1371/journal.pmed.1003435 (PMC7721186; doi:10.1371/journal.pmed.1003435)
Supplement: Attachment — Submitted filename: Response to reviewer comments_2020-Sep-02.docx [file pmed.1003435.s013.docx]

***Requests from the editors:

1.Please include the study population in the title, such as: “Antenatal corticosteroid administration and kindergarten-age child development: a regression discontinuity study in British Columbia, Canada” or similar.***

**Response and change to the manuscript:** We have revised the title as suggested.

***2. Data availability: Thank you for noting that “The data underlying the results can be obtained through PopData BC (***[***www.popdata.bc.ca/***](http://www.popdata.bc.ca/)***)”  If possible, please provide a more direct link or contact address for the specific data set.***

**Response and change to the manuscript:** We have revised the link to be more specific: [www.popdata.bc.ca/dataaccess](http://www.popdata.bc.ca/dataaccess), and included email information for data access staff: [dataaccess@popdata.bc.ca](mailto:dataaccess@popdata.bc.ca). The datasets used in this analysis are listed on the website link. We have also uploaded our statistical code to our Open Science Foundation project repository (<https://osf.io/qxrh4/>).

Our data access statement now reads: “The data underlying the results can be obtained through PopData BC ([www.popdata.bc.ca/dataaccess](http://www.popdata.bc.ca/dataaccess)), contact [dataaccess@popdata.bc.ca](mailto:dataaccess@popdata.bc.ca)”. Statistical code used for our project can be accessed at: <https://osf.io/2evfg/>”

We have also added the link to our statistical code to the statistical analysis section of our Methods: “Statistical code is available at: <https://osf.io/qxrh4/>”

***3. Please structure your abstract using the PLOS Medicine headings (Background, Methods and Findings, Conclusions).***

**Response and change to the manuscript:** The abstract has been restructured as required.

***4. Abstract: In the last sentence of the Objective/Background section, can you please be more specific with the goal of the study, in terms of outcome? “We investigated the safety of antenatal corticosteroid administration practices for child development at kindergarten age.”***

**Response:** We have revised the abstract to reflect that our outcome is a global measure of age-appropriate developmental skills and behaviours.

**Change to the manuscript:** The revised sentence now reads: “We investigated the safety of antenatal corticosteroid administration practices for children’s overall developmental health (skills and behaviours) at early school age”

***5.Abstract: Line 41 (as well as in the Title and throughout text): When referring to “kindergarten age” can you please define or replace with a more universal term or measure of the childrens’ ages?  (e.g. 5 years of age).***

**Response:** We have changed the Title and Text of the manuscript to use the term ‘early school age’ rather than kindergarten. In British Columbia, school intake is based on the calendar year (all children born in a given calendar year begin school in September of the same year), and EDI testing is done the following February. In our cohort, 90% of children were aged 5 at testing, but children with a January or February birthday had already turned 6 at the time of testing. We therefore opted for the term ‘early school age’ rather than ‘age 5 years’. We have retained the term kindergarten in the methods section of the manuscript to describing the timing of the EDI testing, but reworded the sentence for clarity.

**Changes to the manuscript:**

All instances of ‘kindergarten’ changed to ‘early school age’.

Methods, Early Childhood development outcomes section. Revised sentence now reads “British Columbia schools conduct standardized assessments of early school age child development using the Early Development Instrument (EDI) during the first year of school (kindergarten), when children are aged 5-6 years.”

***6. Abstract: Line 44-45 (and in the Introduction at Lines 83-84: Please remove the descriptor "a quasi-experimental design known as" because we feel that most readers will recognize the regression-discontinuity design.***

**Response and change to the manuscript:** The descriptor ‘quasi-experimental design’ has been removed.

***7. Abstract: Methods and Findings: Please include both the 95% CIs and the p values associated with all reported findings.***

**Response and change to the manuscript:**

We respectfully disagree that the addition of p-values would improve our manuscript. Consistent with the guidance of the American Statistical Association (Wasserstein and Lazar, 2016), we provide measures of effect size and precision (i.e., absolute/relative differences with 95% confidence intervals) for all reported findings but do not include p-values because they conflate effect size and precision and may be misinterpreted as probabilities.

From a more practical perspective, consistent with standard practice in RD designs, our models were weighted to assign greater weight to observations closest in gestational age to 34+0 weeks. The addition of these weights invalidated the p-values reported by the model (we used bootstrapping to derive valid estimates of the confidence intervals).

***8. Abstract: Methods and Findings: As the final sentence in this section, please state the main limitation(s) of the study****.*

**Response and change to the manuscript:** We have now added the sentence **“**The regression discontinuity design estimates the effect of antenatal corticosteroid administration practices at the gestational age of the discontinuity, 34+0 weeks, so our results may become less generalisable as gestational age moves further away from this point.**”**

***9. Abstract: Conclusions: Please revise the first sentence to:  “Our study suggests that antenatal corticosteroid administration practices do not appear to affect child development at kindergarten age.”***

**Response and change to the manuscript:** The manuscript has been revised as recommended, although we have modified the language slightly to now read “we found little evidence that antenatal corticosteroid administration practices affect child development”.

***10. Author Summary: At this stage, we ask that you include a short, non-technical Author Summary of your research to make findings accessible to a wide audience that includes both scientists and non-scientists. The Author Summary should immediately follow the Abstract in your revised manuscript. This text is subject to editorial change and should be distinct from the scientific abstract. Please see our author guidelines for more information:*** [***https://journals.plos.org/plosmedicine/s/revising-your-manuscript#loc-author-summary***](https://journals.plos.org/plosmedicine/s/revising-your-manuscript#loc-author-summary)

**Response and change to the manuscript:** We have added the following Author Summary:

**Why was this study done?**

- Antenatal corticosteroids are a medication given to pregnant women before a preterm delivery to help prevent breathing and other sorts of complications in their newborns.
- It has long been recommended that antenatal corticosteroids should be given to women at risk of preterm birth before 34 weeks of pregnancy, but new evidence suggests that newborns of women at risk of preterm birth at 34-36 weeks may also benefit from this medication.
- Because the breathing complications experienced by preterm births at 34-36 weeks are usually less serious in nature and less common, antenatal care providers are increasingly wanting to know if there are any long-term side-effects of antenatal corticosteroids before administering the medication.
- There is limited information on the longer-term safety of antenatal corticosteroids for overall child developmental health.

**What did the researchers do and find?**

- We linked population health and education databases from the province of British Columbia, Canada, to obtain early school age child development test scores in a cohort of children whose mothers were admitted to hospital for delivery between 31 and 36 weeks of pregnancy.
- During the time of our study, most women admitted for delivery before 34 weeks of pregnancy were given antenatal corticosteroids, while most women admitted after 34 weeks were not.
- Since pregnancies admitted for delivery just before and just after 34 weeks are similar in most ways *except* in their chance of receiving antenatal corticosteroids, we compared the child development test scores of children whose mothers were admitted just before and just after 34 weeks of pregnancy to see if scores were lower in children who would have been exposed to antenatal corticosteroids according to clinical practice guidelines.
- We found no difference in child development test scores between children delivered just before and just after 34 weeks of pregnancy.

**What do these findings mean?**

- Our findings suggest that a policy for routine administration of antenatal corticosteroids does not have a negative impact on overall child developmental health.
- Our findings can be used to support clinical counselling on the harms and benefits of antenatal corticosteroid administration at 34-36 weeks of pregnancy.

***11. In text citations: Please place the in-text citation within square brackets before the punctuation mark, like this: [1].***

**Response and change to the manuscript:** The square brackets have been moved to before the punctuation marks as requested.

***12. Introduction: Line 63: Please define the acronym ALPS at first use.***

**Response and change to the manuscript:** The acronym for ALPS (Antenatal Late Preterm Steroids) has now been defined at first use.

***13. Introduction: Line 95: Please revise the sentence: “ The objective of this study was to examine the safety of antenatal corticosteroid administration” to change “safety” to more clearly describe the study outcomes under investigation.***

**Response and change to the manuscript:** The revised sentence now reads “The objective of this study was to examine if antenatal corticosteroid administration practices were linked with children’s overall development (skills and behaviours) at early school age…**”**

***14. Methods: Please specify the manner of informed consent, or if the requirement for consent was waived, and if so by what institution.***

**Response and change to the manuscript:** The need for individual-level informed consent was waived by the BC Children’s Hospital Research Ethics board as part of the ethical approval for the study. We have now expanded our sentence on ethical approval to explicitly state this: “The study was approved by the BC Children’s Hospital Research Ethics Board (#H18-00620), which waived the requirement for individual-level informed consent.”

***15. Methods: Line 148-149: Can you please clarify whether all the children were tested in “kindergarten” or at age 5, or at what ages or range the children were tested?  “The province is tested in 3-year waves, so only approximately one third of children are tested in a given year.”***

**Response:** We have re-written this sentence to improve clarity. In British Columbia, each cycle of EDI testing occurs over a 3 year period. That is, at the end of each testing cycle, all schools in the province have been tested. However, an individual school will only participate once during the 3 year cycle, to decrease the testing burden on teachers and schools. Thus, at a given school, testing is only done on children who started kindergarten during 1 of the 3-year testing cycle and testing is not done on children who started kindergarten during the other two years of the cycle. Testing occurs in the second half of the kindergarten academic year, when teachers are more familiar with the students. The large majority (90% in our cohort) of children are aged 5 when the testing is conducted, but some children in the cohort with an earlier birthday may have already turned 6 at the time of testing (e.g., for testing done in February, children with a January birthday will already have turned 6).

We have clarified our manuscript to explicitly state the age of children at testing, and to better describe the nature of the 3 year testing waves.

**Change to the manuscript:**

Methods, ‘Early childhood development outcomes’ paragraph: timing of testing now described as “during the first year of school (kindergarten), when children are aged 5-6 years”

Methods, ‘Early childhood development outcomes’ paragraph: sentence on provincial testing now reads “Each province-wide testing cycle takes 3 years to complete, meaning that in any given year, only approximately one-third of schools are tested. Students who started school in a year their school was not selected for testing (i.e., 2 of the 3 years within each cycle) do not have EDI scores. However, the EDI scores available (for approximately one third of our birth cohort) provide a representative sample of children from across the province.”

***16. Methods: Line 189: Please replace the term “compliance” with “adherence” where it is used to refer to treatment adherence.***

**Response and change to the manuscript:** This change has been made as requested.

***17. Results: Please provide numbers and percents for missing data within the results or tables.***

**Response:** We now include the number percent of missing data in the tables.

**Changes to the manuscript:** Table 2, insertion of footnote: **“**Total Early Development Index score missing in 50 children (0.9%), Communication skills score in 32 children (0.6%), Emotional maturity score in 68 children (1.2%), Language and cognitive development score in 61 children (1.1%), Physical health and well-being score in 39 children (0.7%), Social competence score in 51 children (0.9%), Developmental vulnerability in 29 children (0.5%), and Special Needs status in 16 children (0.3%).**”**

***18. Results: Line 211: and Table S1: Were there statistically significant differences between included/excluded children/pregnancies?***

**Response:** We intentionally avoided the use of hypothesis testing to compare the characteristics of included *vs.* excluded pregnancies. The goal of this comparison was to determine the potential for selection bias (i.e., the exposure-outcome association in those included in the study was systematically different from the exposure-outcome association in those excluded, making the association in the included sample not generalizable to the whole population). Although the exposure-outcome association cannot be observed in the excluded sample, examining the extent to which the excluded population is similar/different from the included population helps us to speculate on the potential for selection bias. However, conclusions on the potential for selection bias are best informed by examining the clinical importance of the differences between groups rather than statistical significance: with a large enough sample size, an average maternal age of 30.0001 years will be statistically significantly different than an average maternal age of 30 years, despite this difference being clinically irrelevant and unlikely to suggest the potential for selection bias. In a large sample such as our, it is common that differences that are clinically unimportant will be statistically significant. Further, one in 20 hypothesis tests will be significant by chance alone, decreasing their interpretability when comparing multiple different characteristics. We respectfully prefer to retain our focus on the clinical importance of any observed differences between groups, but have revised our language in this section to clarify that comparisons are focused on the clinical importance of any differences.

**Change to the manuscript:** Results, Line 221 “there were no meaningful differences…” now reads “there were no clinically important differences…”

***19. Results: 221-222: Please clarify “meaningful differences” if “statistically significant differences” is meant, and provide 95% CIs and p values in Table 1 supporting this.  “As shown in Table 1, there were no meaningful differences in the characteristics of infants excluded due to lack of EDI testing”***

**Response:** As noted in our response to Editorial Comment 18, we believe that the clinical importance of any differences between groups is more relevant for identifying the potential for bias than the statistical significance of any differences. We agree that the term ‘meaningful differences’ is ambiguous, and revised the sentence to clarify that we examined differences in terms of their clinical importance rather than statistical significance.

**Change to the manuscript:** Results, Line 221 now reads “there were no clinically important differences…”

***20. Results: Line 236-239: Please include p values along with 95% CIs for the result presented here, and please replace “resulted in” to “associated with” to avoid causal impliacations: “Our regression discontinuity model estimated that the higher rates of antenatal corticosteroid administration before 34+0 weeks were associated with a risk ratio for neonatal respiratory morbidity/mortality of 0.81 [95% CI: 0.72 to 0.91], or 5.3 fewer cases per 100 deliveries [95% CI: 2.5 to 8.3]."***

**Response:** Please see our response to Editorial comment #7 above for our response re: adding p-values to reported estimates from our regression discontinuity models.

In general, we agree with the suggestion that causal language should be avoided when reporting the findings of observational studies. However, we believe that in this case, our use of the regression discontinuity design justifies the use of more causal language,

given the caveats we mention in discussing the study design, assumptions and limitations (Hernan, 2018). Regression discontinuity designs have been experimentally shown to provide causal effects consistent with RCTs at the threshold (Cook et al. 2008), which we also provide some evidence for in our analysis, supporting our use of more causal terms such as “effect” or “resulted in”. In addition, our use of causal language is consistent with other quasi-experimental designs published in PLoS Medicine that used similar language (Budu-Aggrey et al. 2019; Jordan et al. 2019; Noyce et al. 2017).

Hernan M. The C-Word: Scientific Euphemisms Do Not Improve Causal Inference From Observational Data. Am J Public Health 2018;108:616-9.

Cook TD, Shadish W, Wong V. Three conditions under which experiments and observational studies produce comparable causal estimates: New findings from within-study comparisons. Journal of Policy Analysis and Management 2008;27:724-50.).

**Change to the manuscript:** None.

***21. Results: Lines 242-249: Please provide both the 95% CIs and p values for all results presented, including the relationships between corticosteroid administration and EDI scores in the discontinuity analysis, and for each of the 5 domains individually.  Please revise this sentence to avoid causal implications: “Figure 4a shows that antenatal corticosteroid administration practices was not associated with total EDI scores (median difference of -0.5 [95% CI: -2.1 to 1.2] at the discontinuity of 34+0 weeks; Table 2).”***

**Response & Change to the manuscript:** Please see our response to Editorial Comment #20 above.

***22. Results: 246-249: Please provide the p values for:  “The point estimate for developmental vulnerability was compatible with 246 a small increase in risk (3.8 excess cases per 100 deliveries [95% CI: -1.3 to 10.0]; RR 1.16 [95% CI: 0.92 to 1.42]), but was not statistically significant.”***

**Response:** Please see our response to Editorial Comment #20 above. We have changed the wording of this sentence to clarify that conclusions about statistical significance were based on the 95% confidence interval.

**Change to the manuscript:** Results, lines 246-249, the phrase ‘…, but were not statistically significant’ now reads ‘…, but the 95% confidence interval provided evidence consistent with a null effect’.

***23.Results: Line 246-249: Please revise to avoid causal implications, and provide p values and 95% CIs with results:  “There was no significant association between antenatal corticosteroid administration practices and special needs designation, although the estimates were imprecise (Figures 4b-c).***

**Response and change to the manuscript:** Please see our response to Editorial Comment #20 above. We have removed causal language and included risk difference and risk ratio estimates with 95% confidence intervals.

**Change to the manuscript:** Results, Line 246-249, revised sentence now reads: “There was no association between antenatal corticosteroid administration practices and special needs designation (excess risk per 100 deliveries -0.5 [95% CI: -4.2, 3.1], RR 0.9 [0.7, 1.4]) ; **Figure 4**).**”**

***24. Results: Line 254-257: Please include both the p values and 95% CIs for these findings: “Using multiple imputation to account for missing EDI results produced estimates for total EDI score, developmental vulnerability, and special needs designation were nearly identical to our primary analyses (-0.5 [95% CI: -2.2 to 1.2], 1.16 [95% CI: 0.95 to 1.4] and 1.0 [95% CI: 0.7 to 1.4], respectively).”***

**Response and change to the manuscript:** Please see our response to Editorial Comment #20 re: p-values.

***25. Discussion: Line 264: Please remove the word “compelling” or replace with “statistically significant” if that is your intended meaning.  Please replace “affected” with “were associated with” to avoid causal implications.***

**Response and change to the manuscript:** We have removed the word compelling as requested. Please see our response to Editorial comment #20 above re: use of causal language.

***26. Discussion: Line 287: Please define the abbreviation ASTECS at first use.***

**Response and change to the manuscript:** we now define the abbreviate ASTECS on first use (Antenatal Steroids for Term Elective Caesarean Section)

***27. Discussion: Line 342: Please replace the term “compliance” with “adherence” where it is used to refer to treatment adherence.***

**Response and change to the manuscript:** We have replaced the term ‘compliance’ with ‘adherence’ as requested.

***28. Discussion: Please include a discussion of the following points:
-Please comment on whether the numbers excluded due to lack of gestational age could contribute to any bias in the analysis.***

**Response:** Our discussion currently includes the sentence “Thus, although excluding births without a day-specific, ultrasound confirmed estimate of gestational age reduced our sample size, we do not believe this was likely to have introduced selection bias.” We have expanded this point to elaborate that the reduced sample size (i.e., the numbers excluded) did not likely introduce bias because the primary consequence of reduced sample size would be to decrease the precision of estimates (i.e., wide 95% confidence intervals), but confidence intervals for our primary study outcome (and most secondary outcomes) had good precision. That is, we estimated that EDI scores were 0.5/50 lower among children more likely to have been exposed to steroids (i.e., delivered <34+0 weeks) than those less likely to have been exposed, with a 95% confidence interval from -2.2 to 1.7. The upper and lower limits of the 95% CI, -2.2 and 1.7 are still clinically unimportant differences, enabling us to confidently conclude that there is no meaningful association between antenatal corticosteroid administration practices and EDI scores. Thus, tightening our confidence intervals further by increasing sample size would not change our conclusions.

**Change to the manuscript:** Results, line 333, insertion of the sentence: **“**The primary consequence of a reduced sample size would be imprecise estimates (i.e., wide 95% confidence intervals), but our primary findings had good precision.**”** *-****Please discuss as a limitation the fact that the assumption that steroids are given on hospital admission cannot be tested.***

**Response:** We have added our inability to empirical test the timing of decision-making on antenatal corticosteroid administration as a limitation.

**Change to the manuscript:** Results, line 341, insertion of the sentence **“**Our assumption that decisions on antenatal corticosteroid administration are made around the time of maternal admission is informed by the clinical experience of co-authors MAS and JL, but cannot be empirically tested in our data.**”** *-****Please include a discussion that the conclusions relate to steroids given to mothers with preterm birth, and do not apply to women giving birth at >34 weeks or at term, and that neurodevelopmental effects may differ between these two groups****.*

**Response:** We agree with this suggestion, and now address this in the Discussion and in the Abstract.

**Change to the manuscript:**

Abstract, Methods and Findings section, insertion of the sentence: “The regression discontinuity design estimates the effect of antenatal corticosteroid administration at the gestational age of the discontinuity, 34+0 weeks, so our results may not be valid for different gestational ages**”**

Discussion, line 352, insertion of the sentences: “Further, a small fraction of women presenting at preterm ages do not deliver preterm (in the ALPS trial, 16% of women presenting to hospital at 34-36 weeks delivered after 36+6 weeks [4]). Our conclusions should not be applied to such pregnancies, and neurodevelopmental effects may differ in this group.**”**

***29. Reporting Checklist: Please ensure that the study is reported according to the STROBE guideline, and include the completed STROBE checklist as Supporting Information. Please add the following statement, or similar, to the Methods: "This study is reported as per the Strengthening the Reporting of Observational Studies in Epidemiology (STROBE) guideline (S1 Checklist)."***

***If STROBE is not the most appropriate guideline, please choose and report according to the most appropriate guidelines here:*** [***http://www.equator-network.org/reporting-guidelines/strobe/***](http://www.equator-network.org/reporting-guidelines/strobe/) ***When completing the checklist, please use section and paragraph numbers, rather than page numbers.***

**Response and change to the manuscript:** We have added the requested statement to the Methods, and now include a STROBE checklist as Supporting Information (S1 Checklist).

***30. Prospective Analysis Plan: Did your study have a prospective protocol or analysis plan? Please state this (either way) early in the Methods section.***

***a) If a prospective analysis plan (from your funding proposal, IRB or other ethics committee submission, study protocol, or other planning document written before analyzing the data) was used in designing the study, please include the relevant prospectively written document with your revised manuscript as a Supporting Information file to be published alongside your study, and cite it in the Methods section. A legend for this file should be included at the end of your manuscript.***

***b) If no such document exists, please make sure that the Methods section transparently describes when analyses were planned, and when/why any data-driven changes to analyses took place. 

c) In either case, changes in the analysis-- including those made in response to peer review comments-- should be identified as such in the Methods section of the paper, with rationale***

**Response:** Our study had a pre-analysis plan, finalized Jan 27, 2020, which was deposited with the Open Science Foundation (<https://osf.io/2evfg/>). We now describe this in the methods and include our pre-analysis plan in the Supporting Information. The analyses reported in our manuscript followed those pre-specified in the plan.

**Change to the manuscripts:** Methods, line 115, insertion of the sentence **“**Analyses were conducted as specified in our prospective analysis plan (S1 Protocol).”

Our pre-analysis plan is now included as supporting information, with an accompanying legend at the end of our manuscript file.

***31.Table 1: Please define abbreviations for BMI and SD in the legend.***

**Response and change to the manuscript:** We have removed these abbreviations from the Table and spell out the words in full.

***32. Table 2: Please define abbreviations for EDI and CI in the legend. Please also present the p values for the risk ratio estimates.***

**Response and change to the manuscript:** We have removed these abbreviations from the Table and spell out the words in full. Please see our response to Editorial Comment #7 re: lack of p-values.

***33. Figure 2 legend: Please change “limited” to “limit”***

**Response and change to the manuscript:** Thank you for bringing this to our attention, this error has been corrected.

***34. Figures 3 and 4: Please specify in the legend that it is the shaded area that indicates the 95% CIs.***

**Response and change to the manuscript:** We now specify in the legends of Figures 3 and 4 (now Figures 2-4) that the shaded area indicates the 95% CIs.

***35. S1 Table 1: Please define SD and BMI in the legend.***

**Response and change to the manuscript:** We have removed these abbreviations from the Table and spell out the words in full.

***36. S2 Table 2: Please define the abbreviations for EDI and CI in the legend.***

**Response and change to the manuscript:** We have removed these abbreviations from the Table and spell out the words in full.

***Reviewer #1: This manuscript reports results of a regression discontinuity study to investigate the effect of corticosteroids on childhood neuro development using data from a population based record linkage study in BC, Canada. The study addresses an important clinical question, the study design is robust and the manuscript itself was a pleasure to read.***

***More detailed comments below:***

***1. Line 185 - below the below - please change.***

**Response and change to the manuscript:** Thank you for bringing this to our attention, we have corrected this typo.

***2. The manuscript states that missing EDI values were imputed - what was the definition of the eligible population for whom imputation was carried out? And what approach was used - further information is needed including a comparison of observed and imputed data. Authors should explicitly state that the main analyses used observed and not MI data and provide the rationale for this****.*

**Response:** Multiple imputation was used in the population of children with an ultrasound-confirmed estimate of gestational age in whom EDI testing was conducted, but scores for a specific test score (e.g., total EDI, a subdomain score, etc) were missing. Because we had problems achieving model convergence in some bootstrap iterations of the multiply imputed datasets, and because the number of children with missing values was small (~1%), we opted to present the complete case analysis for consistency (i.e., we did not want some results based on MI analysis and others on complete case analysis).

We now clarify the population in which multiple imputation was carried out, provide more detail on the multiple imputation procedure itself, and explicitly state that the main analyses used observed not MI data. We also include a comparison of the characteristics of children with vs. without missing EDI data, although we note that it is challenging to interpret because the number of observations with missing values was small (n=50), and cell sizes for many of the characteristics are too small for us to present (due to Data Steward restrictions on presenting cells with low counts for reasons of confidentiality.)

**Changes to the manuscript:**

Table 2, inclusion of a footnote the number and counts of observations with missing EDI data.

Methods, Statistical analysis section, revised description of multiple imputation now reads “In children administered the EDI, we used multiple imputation to account for missing items on the EDI test using multivariate imputation by chained equations with 10 imputed datasets. Models included study outcomes and maternal-fetal characteristics presented in Table 1.”

Supporting Information, inclusion of S4 Table presenting the characteristics of infants with vs without missing data:

| **Maternal-fetal characteristic** | **Missing EDI data**  mean ± standard deviation or n(%) | **Available EDI data**  mean ± standard deviation or n(%) |
| --- | --- | --- |
| n | 50 | 5512 |
| Maternal age (years) | 29± 5 | 31± 6 |
| Nulliparity | 20 (40) | 2753 (50) |
| Smoking in pregnancy | 8 (16) | 706 (13) |
| Pre-pregnancy Body Mass Index (kg/m^2^)^1^ | 24± 5 | 25± 5 |
| Hypertensive disorder of pregnancy^2^ | <10 (-) | 861 (16) |
| Diabetes in pregnancy | 13 (26) | 904 (16) |
| Cesarean delivery | 19 (38) | 2059 (37) |
| Labour induction | 12 (24) | 1407 (26) |
|  |  |  |
| Male fetus | 32 (64) | 3131 (57) |
| Birthweight | 2671± 567 | 2669± 536 |
| Gestational week at admission for delivery^2^  31  32  33  34  35  36 | <10 (-)  6 (12)  <10 (-)  <10 (-)  11 (22)  26 (52) | 196 (4)  297 (5)  401 (7)  662 (12)  1280 (23)  2676 (49) |
| 5-minute Apgar score <7^2^ | <10 (-) | 494 (9) |
| Neonatal respiratory morbidity or mortality^2^ | <10 (-) | 1151 (21) |
| ^1^Among 3932 and 36 women with and without available EDI data with available BMI  ^2^Exact figure not presented due to restrictions on presenting small cell sizes | | |

***3. Figure 1 - it is not clear to me what the full circles indicated. The legend says "coinciding with reduced antenatal corticosteroid administration" - do authors mean the period between 33+4 and 34 weeks?***
**Response and change to the manuscript:** We agree that the full circles were unclear, and have now removed them to simplify the Figure.

***4. Authors note no significant difference in the 1976 trial on line 283. Please can they provide actual estimates to provide further information to readers and avoid the use of arbitrary p-value cut-offs.***

**Response:** We agree with this suggestion and have revised the sentence to provide specific figures and remove the focus on arbitrary p-values.

**Change to the manuscript:** Discussion, line 238, revised sentence now reads: “There were likewise no differences between groups in a follow up of the 1976 US National Heart, Lung, and Blood Institute randomized trial (27.0% of children in the treatment group had an abnormal or suspect McCarthy General Cognitive Index *vs.* 28.4% in the placebo group).”

***5. Maternal pre-pregnancy BMI - authors have these data and it is reassuring to see that the distribution is similar across subsets of the dataset. What is the distribution of BMI across gestational age?***

**Response:** The distribution of BMI across gestational age is shown in Figure S3 (top row, right hand column). This plot shows that BMI was uniform across gestational age between 31+0 and 36+6 weeks.

**Change to the manuscript:** None.

***6. How many child deaths were there (assigned to 0 EDI)?***

**Response:** There were 142 deaths in our cohort, which included 46 neonatal deaths and 96 post-neonatal deaths. These infants were assigned the lowest observed EDI score. We now specify this in the Results section of the manuscript.

**Change to the manuscript:** Results, Line 221. Insertion of the phrase “Of these… were child deaths (46 neonatal deaths and 96 post-neonatal deaths).”

***7. Line 297 - authors state that observational studies are at increased risk of confounding bias which is absolutely true. But I would point out that the issue is a bit ore nuanced. The current study also analyses observational data - so I would argue that it is the analytical approach that differs in likelihood of confounding bias and would urge authors to use more nuanced language to clarify this.***

**Response:** This point is well taken, and we have revised the sentence to clarify that the analytic approach rather than the observational nature of the data is what limits conclusions that can be drawn from this paper.

**Change to the manuscript**: Discussion, line 297, revised sentence now reads “However, the majority of studies in the meta-analysis were observational studies that used conventional regression adjustment to control for confounding.”

***8. Table S2 - please add the ref category, i.e. is a positive difference indicate a higher score in kids treated or untreated. The difference in the total score is somewhat odd - worth double checking? Also, adding the Ns included in each column would be helpful. Finally, there are no details on the criteria/how the model selected optimal window is identified.***

**Response:** We have now clarified that the coefficients should be interpreted as the effects of the exposed (i.e., admission prior to 34+0 weeks) compared with the unexposed (admission at or beyond 34+0 weeks), double-checked the total score (it is correct), added column Ns, and provided details on how the model selected optimal window is defined.

**Changes to the manuscript:** S2 Table:

Insertion of Table header: “Estimated effect of corticosteroid administration practice (<34 weeks’ gestation vs. reference of ≥34 weeks)”

Insertion of column Ns (n=5562, 2662, and 1069 for 21,14, and 7 days, respectively)

Insertion of footnote for model-selected optimal window results: “Optimal window (i.e., bandwidth) selected using the default ‘mserd’ option in the Stata 16 command rdrobust, which uses one common mean squared error (MSE)-optimal bandwidth selector for the regression discontinuity treatment-effect estimator.”

***Reviewer #2: The impact of antenatal corticosteroids on later neurodevelopmental outcome is both topical and important. The study addresses this question using an interesting design in a very large and fairly recent linked cohort. The linkage performed and the statistical analysis are impressive. The conclusion that administering steroids at ≤34 weeks does not result in worse neurodevelopmental outcome is perhaps not surprising, but reassuring nonetheless. I have some general and some more specific comments for the authors:

1. The issue of using gestation of admission as a proxy for gestation of steroid administration is a bit more complex than presented. I appreciate that it is not possible to know when steroids were actually given during an admission, and that the gestation at admission may be the best proxy possible for the time of steroid administration. However, it is not clear that the actual gestation of delivery is known. If this is available, then it would be useful to present some measures of the variance in interval between admission gestation and delivery gestation. There are two points connected with this. Firstly, the gestation of delivery may be more impactful on the neurodevelopmental outcome than the gestation of steroid administration, and the overall conclusions may be affected by the noise introduced by the variance introduced in steroid-to-delivery interval. For some women, for example those admitted to monitor bleeds from a low-lying placenta, there may be a very long interval between admission and delivery, whereas others will arrive and deliver very quickly. Women who arrive and deliver very quickly also present a problem to the analysis, in that the steroids may not have reached efficacy if the administration to delivery interval is short (this is known wrt lung maturation). The admission to delivery interval would perhaps be expected to decrease with increasing gestational age as the risk/benefit ratio of intervening changes - i.e. a 36 week baby may be delivered at first suspicion of a problem, whereas a 31 week baby may provoke more concern and a greater tendency to try to hold off delivering, for example until the germinal matrix is formed. I note that at least a quarter of the cohort were induced or delivered by CS (of which a proportion will be elective or semi-elective). If this is the case, then the error associated with the admission-to-delivery interval will change across the analysis.***

**Response:** We agree that gestational age at delivery is likely a stronger predictor of adverse neurodevelopmental outcomes than gestational age at admission. Our primary consideration in selecting the gestational age variable, however, was not its ability to predict the outcomes, but rather, its ability to maximize the discordance in antenatal corticosteroid exposure status (i.e., create two gestational age categories that had highest vs. lowest rates of antenatal corticosteroid administration). As decisions on antenatal corticosteroid administration are most commonly made at the time of admission, the use of gestational age at admission, rather than delivery, is the preferable variable for these analyses. Error would not be introduced by the difference between the admission date and delivery date (i.e., the admission-to-delivery interval), but rather, if the decision-making around antenatal corticosteroid administration happened at a gestational age other than admission. Although based on the clinical experience of the two authors who are practicing obstetricians in British Columbia (JL and MAS), this assumption is difficult to verify quantitatively, and we have added this point to our limitations section.

We conducted new analyses to examine admission-to-delivery interval, labour induction, and iatrogenic delivery (i.e., by either induction or pre-labour Cesarean) by gestational age. As speculated by the reviewer, the admission-to-delivery interval changes across gestational age, with pregnancies admitted at younger gestational ages more likely to be managed expectantly than those admitted closer to term. Likewise, the proportion of births delivered following induction increases as term approaches. However, these patterns would only introduce bias to the regression discontinuity design estimates if they do not change smoothly across gestational age but rather, had a pronounced shift at the point of the discontinuity. We have now added three additional graphs to Figure S3 (now Figure S5) which plot labour induction, iatrogenic delivery, and admission-to-delivery interval by day of gestation, and show that these variables change smoothly across gestation.

**Changes to the manuscript:**

Discussion, limitations section, insertion of the sentence**: “**Our assumption that decisions on antenatal corticosteroid administration are made around the time of maternal admission is informed by the clinical experience of co-authors MAS and JL, but cannot be empirically tested in our data.**”**

S3 Figure (now S5 Figure): insertion of three new graphs showing iatrogenic delivery, labour induction, and admission-to-delivery interval by gestational age.

***2. The second point is that if the hypothesis is that the gestation when the brain is first exposed to steroids is the determinant of long-term outcome (which is a reasonable hypothesis and hence the use of admission dates), rather than the timing of delivery/admission, then the statement in lines 137-138 that steroid administration prior to delivery admission is not an important issue is difficult to reconcile. Presumably the risk of having had steroids during a prior admission increases with babies admitted at later gestations, as a function of time-at-risk?***

**Response:** This is an excellent point. It is important to clarify that our regression discontinuity design is not evaluating the effect of gestational age at administration per se, it is evaluating the effect of current administration practices (i.e., higher likelihood of exposure by virtue of being admitted prior to 34+0 weeks vs lower likelihood of exposure by virtue of being admitted post 34+0 weeks). As a result, an infant admitted for the delivery admission at 33+6 weeks who received steroids on a previous admission at 33+2 weeks (so was not documented as having received steroids in the BC Perinatal Data Registry variable) would still correctly be classified as having a higher likelihood of exposure.

The concern would arise in pregnancies admitted after 34+0 weeks that had received steroids on a previous antenatal admission, but would be incorrectly classified as having a low likelihood of exposure by virtue of being admitted for the delivery admission after 34+0 weeks. In the ALPS trial of antenatal corticosteroids at late preterm ages (34-36 weeks), 3269 pregnancies of 24,333 screened for trial eligibility (13.5%) had been administered corticosteroids prior in the pregnancy (*N Engl J Med* 374;14;1311). If rates are similar in the British Columbia population, this could potentially have introduced bias to our study. However, this bias would also have been expected to affect our analysis of neonatal respiratory morbidity/mortality. Our finding of a protective effect for that association comparable in magnitude to those previously reported in randomized trials suggests that the bias is small in magnitude (as these analyses would have been affected by the same bias).

We have added a discussion of this point to our limitations section, and clarify in the introduction that our study is analogous to a randomized trial where participants are randomized to a policy of routine antenatal steroids vs. no routine antenatal steroids.

It is challenging to speculate on whether the risk of having been given steroids on a previous admission increases with advancing gestational age. We agree that it is plausible that the chance of a previous admission could increase with advancing gestational age (i.e., as a function of increased at-risk time), but one can also speculate that at later gestational ages, the chance that an admission will result in delivery increases (i.e., at late preterm ages, the benefits of expectant management are fewer than at younger preterm ages, so pregnancies with an indication for delivery at late preterm ages (e.g., maternal hypertension, premature rupture of membranes, fetal growth restriction) are more likely to simply be delivered rather than discharged and readmitted later).

**Change to the manuscript:** Discussion, Limitations section, insertion of the sentences: “Further, in the ALPS trial [4], 3269 of 24,333 (13.5%) pregnancies screened for eligibility at 34-36 weeks had received antenatal corticosteroids earlier in pregnancy. If a sizeable fraction of infants in our cohort admitted for delivery at 34-36 weeks had also received steroids earlier in pregnancy, this could introduce bias to our findings (since our analysis assumed that infants admitted after 34 weeks had a low likelihood of exposure to antenatal corticosteroids). It is reassuring, however, that our estimates of effect for neonatal respiratory morbidity/mortality were compatible with those reported from randomized trials [26], suggesting the magnitude of any such bias is likely small.”

Introduction, lines 126-128, insertion of the sentence “In other words, we mimicked a pragmatic randomized trial in which one group had a policy to receive the intervention (antenatal corticosteroids), and the other group did not, but both groups are otherwise expected to be similar.”

***3. Given that only 50-60% of the cohort at gestations ≤34 weeks actually had steroids, this seems an important point to draw out and discuss. Why is this percentage so low? Was the national guidance regarding steroid administration changed during the study period? Could a sensitivity analysis be performed introducing a dummy variable for babies actually given steroids?***

**Response and change to the manuscript:** We do not believe that only 50-60% of the cohort≤34 weeks received corticosteroids. Rather, this observed rate likely reflects under-reporting of true administration rates because of a known limitation of the BC Perinatal Data Registry variable for antenatal corticosteroid administration. The BC Perinatal Data Registry variable for antenatal corticosteroids is defined as ‘Steroids for lung maturation received during the delivery admission’, with instructions for coders to indicate whether ‘mother received steroid medication during the inpatient delivery episode of care, or for transport.’ However, this means that coders do not abstract information on antenatal corticosteroid administration that occurred in a hospitalization prior to the delivery admission (e.g. a mother presents to hospital with threatened preterm labour, is given corticosteroids, is discharged without delivering, but is readmitted 2 days later for delivery). Likewise, administration may not be abstracted if it is unclear from the chart *when* the steroids were given. This known limitation of the BC Perinatal Data Registry variable is being corrected in an upcoming Registry revision.

We have good reason to believe that the true administration rates are higher: following a previous report on use of antenatal corticosteroid use in British Columbia (*J Obstet Gynecol Canada* 2012;34:842-848) that identified similarly lower than expected administration rates, Perinatal Services BC, the organization responsible for maintaining the BC Perinatal Data Registry, conducted an internal audit of charts to identify reasons for the low administration rates (corresponding author JAH was a member of the Perinatal Services BC Surveillance Team that planned and reviewed the results). This audit was done by a maternal-fetal medicine subspecialist and an obstetrical nurse, and included 75 charts of randomly-sampled preterm births from two different institutions in British Columbia. A key finding was that many of the infants that the Data Registry had classified as not having been given antenatal corticosteroids had in fact been given them, but just not during the delivery admission, as speculated above. It was estimated that true administration rates were closer to 70-80% (Dr Gerald Marquette, Perinatal Services BC, personal communication). However, as this internal analysis is unpublished, and data were not collected for publication purposes, we do not feel comfortable including the findings in our report.

A sensitivity analysis based on actual receipt of steroids would lose the benefits of improved control for confounding obtained through the regression discontinuity design.

We have inserted an additional sentence clarifying why the known under-documentation in the corticosteroids variable is not believed to have to introduced bias.

**Changes to the manuscript**: Methods, description of antenatal corticosteroid variable now reads (new text underlined): “Antenatal corticosteroid administration is available in the BC Perinatal Data Registry as a binary variable indicating the administration of corticosteroids during the delivery admission for the purpose of fetal lung maturation, but not administration during an antenatal hospitalization that did not result in delivery, therefore underestimating overall administration. This known under-documentation did not affect our analyses, which used the gestational age discontinuity as the exposure, not corticosteroid status *per se*. That is, we relied instead on the assumption that antenatal corticosteroid administration recommendations were largely followed in practice, such that pregnancies admitted just before 34 weeks had a much higher change of being exposed to antenatal corticosteroids than those admitted just after (which we observed in our data).”

We are willing to add in personal communication information supporting the likely under-documentation if deemed useful, but prefer to avoid inclusion of unpublished data if possible.

***4. Can the authors further describe the assumption that the impact of gestation of delivery on later neurodevelopmental outcome is smooth - is there an assumption of linearity or any specified form?***

**Response:** We have expanded our description of the assumptions associated with our modelling of gestational age.

**Change to the manuscript:** Methods, line 200, insertion of the sentence, “That is, all models assumed that risk of adverse neurodevelopmental outcomes changed smoothly across gestation (i.e., in the absence of an effect of corticosteroids at 34+0 weeks); the use of the quadratic term assessed whether this change occurred in a non-linear manner.”

***5. Introduction, lines 63-69: it is not really clear how this manuscript, although interesting with regards to the neurodevelopmental impact of steroids given prior to 34 weeks, adds to the body of evidence regarding later steroid administration. Is exposing the fetal brain to steroids at any stage of development equivalent? On further reading, I realise that this point is addressed at the very end of the limitations section. However, it is an odd way to commence the manuscript, given that the data don't address this question.***

**Response:** The regression discontinuity design provides an estimate of treatment effect at the point of the discontinuity (here, 34+0 weeks). The further away from the discontinuity, the less generalizable the findings. As the current controversy over antenatal corticosteroid administration is focused primarily on late preterm ages (34-36 weeks), we believe that our findings are highly relevant to this debate, particularly those presenting at the earlier ages of the late preterm range (i.e., those presenting 34 weeks). We agree, however, that interpretation should be more and more cautious as gestational age moves farther away from 34+0, and have expanded our discussion of this limitation to emphasize this point. We have also added a sentence to the Statistical analysis section of the Methods reiterating that the regression discontinuity design estimates the causal effect of an intervention at the point of the discontinuity.

**Changes to the manuscript:**

Abstract, Methods and Findings section, insertion of the sentence: **“**The regression discontinuity design estimates the effect of antenatal corticosteroid administration practices at the gestational age of the discontinuity, 34+0 weeks, so our results may become less generalizable as gestational age moves further away from this point.**”**

Discussion, limitations section, line 350, revised sentence now reads (new text underlined) **“**Finally, the regression discontinuity design only allows inference on the effects of antenatal steroid administration at the point of the discontinuity: 34+0 weeks.^14^ It is possible that the effects of antenatal corticosteroids on child neurodevelopment differ according to gestational age (especially at term), and the generalisability of our findings may decrease as gestational age moves farther from 34+0 weeks.**”**

Methods, Statistical analysis section, insertion of the sentence “In other words, the regression discontinuity design estimates the causal effect of the intervention at the point of the discontinuity (here, 34+0 weeks).”

***6. Methods: Although the linked cohort is very large, the number of babies born preterm per day for whom there is linked data actually works out fairly small at earlier gestations. Was any analysis to investigate power performed?***

**Response:** Yes, we performed simulation studies at the study design stage to ensure that we would have sufficient statistical power. Using a fixed sample size of eligible preterm births in the BC Perinatal Data Registry based on pilot data (and assuming EDI scores would only be available for one-third of infants due to 3 year testing waves), the gestational age distribution of births within this age window from our pilot data, the mean and standard deviation of EDI scores among preterm births (*Pediatrics* 2016;138:e20162002), and the underlying association between gestational age and child development scores (*Archives of Disease in Childhood* 2000;83:25-30), we created multiple simulated datasets and ran regression discontinuity analyses to assess our power to detect different treatment effects (i.e., magnitude of the difference in EDI scores before and after the 34+0 week discontinuity). From this, we estimated that we would have >90% power to detect an effect size of 0.2 standard deviations or greater, suggesting we would be able to identify even small effects of antenatal corticosteroid administration. Even if we conservatively restricted to the two weeks before and after 34+0 weeks’ gestation, we had >80% power to detect an effect size of 0.3.

**Change to the manuscript:** We opted not to include our sample size calculation in the manuscript for conciseness, as our primary analyses had good statistical precision (demonstrating that under-power/small sample size was not a concern), but are willing to include this paragraph if requested.

***7. The breadth and detail of the EDI data available are impressive and comprehensive***

**Response and change to the manuscript:** Thank you, we agree that the EDI data are a strength of the study.

***8. Lines 124-127: this seems like an important point to provide a little more information on, as this method has been applied for the majority of the cohort, 2000-2008. Given that the discontinuity is based on a single day difference, could the authors add to the paper the number of observations potentially mis-categorised by this, i.e. the number of patients born prior to 2008, within weeks 33/34?***

**Response:** There were 569 (10.2%) infants born prior to 2008 who were admitted for the delivery admission between 33+0 and 34+6 weeks. In order to assess the potential for bias from including of these potentially misclassified infants, we conducted a sensitivity analysis restricting to births admitted after April 1, 2008. In these models, confidence intervals were wider (reflecting the smaller sample size), but conclusions were unchanged.

**Changes to the manuscript:**

Methods, Sensitivity analyses section, insertion of the sentence: **“**We restricted analyses to children admitted for delivery after April 1, 2008.**”**

Results, Sensitivity analyses section, insertion of the phrase ‘…or restricting to admissions post-April 1, 2008’ to the sentence “Changing the gestational age window of births included in the analyses, or restricting to admissions post-April 1, 2008, did not meaningfully impact our conclusions (**Table S2 and S3**).”

Supporting Information, insertion of new S3 Table (see next page):

**S3 Table.** Effect of restricting to births admitted after April 1, 2008 when estimating the effect of antenatal corticosteroid administration practices among 5562 children in British Columbia, Canada.

| **Outcome** | **Estimated effect of corticosteroid administration practice (<34 weeks’ gestation vs. reference of ≥34 weeks)** | |
| --- | --- | --- |
|  | **Absolute difference in median scores [95% confidence intervaI]** |  |
| Total Early Development Index score (/50) | 2.1 [-1.5, 4.0] | - |
| Communication skills score (/10) | 0.2 [-1.3,2.0] | - |
| Emotional maturity score (/10) | 0.0 [-0.6, 0.7] | - |
| Language and cognitive development score (/10) | 0.6 [0.4, 1.0] | - |
| Physical health & well-being score (/10) | 0.0 [-0.4, 0.5] | - |
| Social competence score (/10) | 0.3 [-0.4, 1.0] | - |
|  |  |  |
|  | **Excess cases per 100 births [95% confidence interval]** | **Risk ratio [95% confidence interval]** |
| Developmentally vulnerable | 0.9 [-8.7,11.5] | 1.0 [0.7, 1.4] |
| Special needs designation | 0.4 [-5.7,7.4] | 1.0 [0.5, 1.8] |

***9. Line 157-158: is this a score of 1 or 0/10 in each domain? Or is this a cohort-specific or population-specific 10th centile?***

**Response:** We have revised our description of ‘developmentally vulnerable’ to indicate that this is based on the population 10^th^ percentile (i.e., external to our cohort).

**Change to the manuscript: Methods, Line 157-58 now reads:** ‘developmentally vulnerable’ (<10^th^ population percentile on ≥2 domains).

***10. Line 169: were all early neonatal deaths included here? Or only those linked to respiratory morbidity?***

**Response:** we now clarify that all neonatal deaths were included.

**Change to the manuscript:** Methods, Neonatal outcomes, Line 169, revised phrase now reads “all-cause death<28 days after birth”

***11. Line 226: it would be better to replace this single figure of 45% points with the average before v. after***

**Response:** We have included the before and after values in addition to the difference of 45%.

**Change to the manuscript:** Results, line 226, insertion of the text “47% vs. 2%)”

***12. Line 341: I had understood Figure 1 to be a schematic of hypothetical data? How does it show that a discontinuity exists in the actual data? Can the labelling be a bit more specific about what Figure 1 represents - if it really is just an example of what a regression discontinuity analysis looks like, then it might be better as a supplementary figure, and the much more interesting figure S2 included in the main manuscript instead.***

**Response:** Yes, it is a schematic of hypothetical data intended primarily for readers less familiar with the design, and does examine the presence of a discontinuity in our actual data. We agree with the suggestion to move it to the Supporting Information section. As suggested by Reviewer 3, we have made figures 4a-c independent figures to emphasize their importance, so have opted to retain Figure S2 in the Supporting Information for now.

**Changes to the manuscript:** Figure 1 (hypothetical schematic) moved to the Supporting Information (S2 Figure); Figures 4a-c now standalone figures (Figures 2-4 in revised version).

***13. Table 1: inducing a quarter of preterm fetuses is interesting. Can the authors comment on what gestation induction might have been considered/attempted? These are presumably mainly 35-36 weekers. Do you have data on spontaneous v. iatrogenic delivery overall? Could this be added to the useful and well-presented figure S3?***

**Response and change to the manuscript:** Thank you for this suggestion. During the study period, the overall induction rate in British Columbia was approximately 20% (<http://www.perinatalservicesbc.ca/Documents/Data-Surveillance/Reports/PHR/PHR_BC_Deliveries_201314.pdf>); the induction rate of approximately 25% in our cohort is slightly higher, but not unexpectedly so given the high-risk nature of preterm deliveries. We agree that it would be interesting to show the patterns of iatrogenic delivery (delivery following labour induction or pre-labour cesarean delivery) by gestational age, and have added this to figure S3.

**Change to the manuscript:** insertion to figure S3 of a plot showing iatrogenic delivery by gestational age:

***14. Implications: I think that the first statement here is a really useful and important finding of the study. I'm much less convinced by the extrapolation inherent in the second statement, and I would think about re-working the introduction and discussion more towards the reassurance the study provides regarding neurodevelopmental implications prior to 34 weeks. I realise this may seem a little less 'high impact' - but it is important in its own right.***

**Response:** Our regression discontinuity design estimates the effect of antenatal corticosteroid administration practices at 34+0 weeks (i.e., at the point of the discontinuity). As the late preterm period begins at 34+0 weeks, we believe that it is very reasonable to conclude that our findings may be useful for informing care decisions at late preterm ages. We agree, however, that interpretation should be more and more cautious as gestational age from 34+0 increases (as outlined in Reviewer 2 comment 5 above), and have expanded our discussion of this limitation to emphasize this point. We also reiterate in the statistical analysis section of our Methods that the regression discontinuity design estimates the causal effect of the intervention at the point of the discontinuity (here, 34+0 weeks).

**Change to the manuscript:**

Abstract, Methods and Findings section, insertion of the sentence: **“**The regression discontinuity design estimates the effect of antenatal corticosteroid administration practices at the gestational age of the discontinuity, 34+0 weeks, so our results may become less generalisable as gestational age moves further away from this point.**”**

Discussion, limitations section, line 350, revised sentence now reads (new text underlined) **“**Finally, the regression discontinuity design only allows inference on the effects of antenatal steroid administration at the point of the discontinuity: 34+0 weeks.^14^ It is possible that the effects of antenatal corticosteroids on child neurodevelopment differ according to gestational age (especially at term), and the generalisability of our findings may decrease as gestational age moves further from 34+0 weeks.**”**

Methods, Statistical analysis section, insertion of the sentence “In other words, the regression discontinuity design estimates the causal effect of the intervention at the point of the discontinuity (here, 34+0 weeks).”

***Reviewer 3***

***Points of detail***

***1. Page 6 I am afraid I did not grasp the issue of the 3–year waves. Does this mean that every third year all children in a three year age band are tested and then two years elapse before the next wave? That does not seem to ﬁt with the rest of the text. Supplementary Figure 1 suggests something diﬀerent too.***

**Response:** We agree that this issue was not well explained, and have re-written our explanation of the testing process to improve clarity. Please see our response to Editorial comment #15.

**Change to the manuscript: Methods, Early Child Development outcomes paragraph now reads: “**Each province-wide testing cycle takes 3 years to complete, meaning that on any given year, only approximately one-third of schools are tested. Students who started school in a year their school was not selected for testing (i.e., 2 of the 3 years within each cycle) do not have EDI scores. However, the EDI scores available (for approximately one third of our birth cohort) provide a representative sample of children from across the province.**”**

***2. Page 8 Presumably the kernel is chosen to fall to zero at the limits of the data?***

**Response:** Yes, this is correct. The observations at the limit of the data in our study (31+0 and 36+6 weeks) have a near-zero weight, and the observations one day earlier/later than our data range (30+6 and 37+0 weeks) would have a weight of zero if they were included in our analytic cohort. We now explicitly state this in our methods.

**Change to the manuscript:** Methods, line 194 now reads **“**such that observations closest to the discontinuity were assigned the greatest weight analytically and weights fell to near-zero at the limits of the data**”**

***3. Page 8 Presumably a quadratic term for age is meant?***

**Response and change to the manuscript:** Yes; the term ‘squared’ has now been replaced with ‘quadratic’.

***4. Page 8 The multiple imputation (MI) model is not really described here and could use a bit more detail about the variables in the model, which variables needed imputing, how many sets were generated and how much was missing for instance. This could go into the supplement if the authors feel it would break up the ﬂow here.***

**Response:** We agree that more information should be provided on our use of multiple imputation. Our revised manuscript now includes the number and percent of observations with missing EDI data, and greater details on the multiple imputation methods used. We now also include comparison of observations with and without missing EDI data in the Supporting Information, but note that it is challenging to interpret because the number of observations with missing values was small (n=50), and cell sizes for many of the characteristics are too small for us to present (due to Data Steward restrictions on presenting cells with low counts for reasons of confidentiality.)

**Changes to the manuscript:**

Table 2, inclusion of a footnote the number and counts of observations with missing EDI data.

Methods, Statistical analysis section, revised description of multiple imputation now reads “In children administered the EDI, we used multiple imputation to account for missing items on the EDI test using multivariate imputation by chained equations with 10 imputed datasets. Models included study outcomes and maternal-fetal characteristics presented in Table 1.”

Supporting Information, inclusion of S4 Table presenting the characteristics of infants with vs without missing data:

| **Maternal-fetal characteristic** | **Missing EDI data**  mean ± standard deviation or n(%) | **Available EDI data**  mean ± standard deviation or n(%) |
| --- | --- | --- |
| n | 50 | 5512 |
| Maternal age (years) | 29± 5 | 31± 6 |
| Nulliparity | 20 (40) | 2753 (50) |
| Smoking in pregnancy | 8 (16) | 706 (13) |
| Pre-pregnancy Body Mass Index (kg/m^2^)^1^ | 24± 5 | 25± 5 |
| Hypertensive disorder of pregnancy^2^ | <10 (-) | 861 (16) |
| Diabetes in pregnancy | 13 (26) | 904 (16) |
| Cesarean delivery | 19 (38) | 2059 (37) |
| Labour induction | 12 (24) | 1407 (26) |
|  |  |  |
| Male fetus | 32 (64) | 3131 (57) |
| Birthweight | 2671± 567 | 2669± 536 |
| Gestational week at admission for delivery^2^  31  32  33  34  35  36 | <10 (-)  6 (12)  <10 (-)  <10 (-)  11 (22)  26 (52) | 196 (4)  297 (5)  401 (7)  662 (12)  1280 (23)  2676 (49) |
| 5-minute Apgar score <7^2^ | <10 (-) | 494 (9) |
| Neonatal respiratory morbidity or mortality^2^ | <10 (-) | 1151 (21) |
| ^1^Among 3932 and 36 women with and without available EDI data with available BMI  ^2^Exact figure not presented due to restrictions on presenting small cell sizes | | |

***5. Page 9, Figure 3 I am not sure why it is necessary to highlight the points near the discontinuity especially as that does not seem to have been done in Figure 4.***

**Response and change to the manuscript:** We have now removed the highlighted points near the discontinuity.

***6. Page 10 The report of the MI here suggests that what happened was that there was item missing on the EDI and that was what was imputed. Is that the case?***

**Response:** Yes, that is correct. We have now provided an expanded explanation of our use of multiple imputation that clarifies this point.

**Change to the manuscript:** Methods, Statistical analysis section, revised description of multiple imputation now reads “In children administered the EDI, we used multiple imputation to account for missing items on the EDI test using multivariate imputation by chained equations with 10 imputed datasets. Models included study outcomes and maternal-fetal characteristics presented in Table 1.”

***7. Pages 12 and 13 I thought the strengths and limitations section was very fair and balanced****.*

***Point of more substance***

***8. The authors present their results in the sequence: description, details of intervention, eﬀect of morbidity and mortality, eﬀect on development, sensitivity analyses. When I ﬁrst read the results section and when I ﬁrst looked at Figures 1 to 4 I almost missed the main point of the article, the point which is summarised in the ﬁrst sentence of the discussion. Is there some way of re–structuring this to make the main message more prominent? For instance the analysis of morbidity and mortality is more like a sensitivity analysis to conﬁrm for us that the method would be capable of conﬁrming a well known result. Figure 3 is much more prominent than Figure 4 as 4 contains small sub–ﬁgures, perhaps move 3 into 4 or make 4 three ﬁgures? I would also make Figure 1 more schematic as the unwary reader might think it shows actual results. I would label the y–axis ‘Risk of event’, replace the red text with ‘Intervention’, ‘No intervention’ and replace ‘eﬀect of corticosteroids’ by ‘eﬀect of intervention’. I agree that the absence of axis tick marks rather gives the game away and when printed the caption will be with the ﬁgure not pages away.***

**Response and change to the manuscript:** Thank you for these suggestions. We have now moved Figure 1 (hypothetical schematic of the RD design) & Figure 3 (neonatal outcomes) to the Supporting Information, and made each of Figures 4a-b stand alone figures (now Figures 2-4) to better emphasize their importance.
